# 13CFLUX - third-generation high-performance engine for isotopically (non)stationary ^13^C metabolic flux analysis

**DOI:** 10.1093/bioinformatics/btaf630

**Published:** 2025-11-18

**Authors:** Anton Stratmann, Martin Beyß, Johann F Jadebeck, Wolfgang Wiechert, Katharina Nöh

**Affiliations:** Institute of Bio- and Geosciences, IBG-1: Biotechnology, Forschungszentrum Jülich GmbH, 52425 Jülich, Germany; Computational Systems Biotechnology (AVT.CSB), RWTH Aachen University, 52062 Aachen, Germany; Institute of Bio- and Geosciences, IBG-1: Biotechnology, Forschungszentrum Jülich GmbH, 52425 Jülich, Germany; Computational Systems Biotechnology (AVT.CSB), RWTH Aachen University, 52062 Aachen, Germany; Institute of Bio- and Geosciences, IBG-1: Biotechnology, Forschungszentrum Jülich GmbH, 52425 Jülich, Germany; Computational Systems Biotechnology (AVT.CSB), RWTH Aachen University, 52062 Aachen, Germany; Institute of Bio- and Geosciences, IBG-1: Biotechnology, Forschungszentrum Jülich GmbH, 52425 Jülich, Germany; Computational Systems Biotechnology (AVT.CSB), RWTH Aachen University, 52062 Aachen, Germany; Institute of Bio- and Geosciences, IBG-1: Biotechnology, Forschungszentrum Jülich GmbH, 52425 Jülich, Germany

## Abstract

**Summary:**

^13^C-based metabolic flux analysis is a cornerstone of quantitative systems biology, yet its increasing data complexity and methodological diversity place high demands on simulation software. We introduce 13CFLUX(v3), a third-generation simulation platform that combines a high-performance C++ engine with a convenient Python interface. The software delivers substantial performance gains across isotopically stationary and nonstationary analysis workflows, while remaining flexible to accommodate diverse labeling strategies and analytical platforms. Its open-source availability facilitates seamless integration into computational ecosystems and community-driven extension. By supporting multi-experiment integration, multi-tracer studies, and advanced statistical inference such as Bayesian analysis, 13CFLUX provides a robust and extensible framework for modern fluxomics research.

**Availability and implementation:**

Sources and containers are provided at https://jugit.fz-juelich.de/IBG-1/ModSim/Fluxomics/13CFLUX, and scripts to replicate results in the supplementary data at https://github.com/JuBiotech/Supplement-to-Stratmann-et-al.-Bioinformatics-2025.

## 1 Introduction

Intracellular metabolic reaction rates (fluxes) at steady state are crucial for understanding cellular metabolism quantitatively. To determine fluxes in living cells, a computational approach is required, where unknown fluxes are inferred from data using metabolic models. Of all fluxomics techniques, ^13^C-based metabolic flux analysis (MFA) is considered the most informative. ^13^C-MFA utilizes data from isotope labeling experiments (ILE) and external rate measurements to estimate fluxes and their uncertainties within the context of metabolic networks (Niedenführ *et al.* 2015). This technology is well-established in metabolic engineering, bioprocess engineering, and health research, enabling the characterization of microbes (Long and Antoniewicz 2019), plants (Xu *et al.* 2022), and mammalian cells (Hogg *et al.* 2023).

Advances in experimental-analytical techniques have led to various extensions of ^13^C-MFA. For instance, integrating data from multiple isotopically stationary (IST) ILEs, either from the same or different analytical platforms (Rahim *et al.* 2022), or the integration with genome-scale models (McCloskey *et al.* 2016b), has enhanced the information gain. Developing case-specific labeling strategies (Borah Slater *et al.* 2023, Mitosch *et al.* 2023) and mass spectrometry methods (McCloskey *et al.* 2016a, Kappelmann *et al.* 2019) have further expanded the scope of ^13^C-MFA (Gopalakrishnan and Maranas 2015, McCloskey *et al.* 2016b), while the miniaturization and automation of ILEs on robotic platforms have improved their economic feasibility (Fina *et al.* 2023). When combined with rapid quenching protocols, the label incorporation into intracellular metabolites is trackable in small-scale bioreactor systems such as the BioLector (Nießer *et al.* 2022), paving the way for a broader applicability of isotopically nonstationary (INST) ^13^C-MFA (Nöh *et al.* 2006). These developments have broadened the application of ^13^C-MFA, but they have also raised the bar for the robustness and reliable performance of the computational ^13^C-MFA toolset.

The evaluation workflow of ^13^C-MFA consists of three main steps: experimental design, parameter fitting/optimization, and statistical analysis/uncertainty quantification (Zamboni *et al.* 2009, Long and Antoniewicz 2019). To address these steps, numerous methodological developments have emerged, including robust algorithms for ILE design (Beyß *et al.* 2021), fast nonlinear optimization (Lugar and Sriram 2022), and high-throughput machine learning strategies (Wu *et al.* 2022). The classical statistical toolkit has been enriched by Bayesian approaches (Theorell *et al.* 2017, Backman *et al.* 2023), allowing researchers to address existing questions and tackle new ones (Theorell *et al.* 2024). Consequently, the configuration of ^13^C-MFA workflows has become increasingly diverse and computationally demanding. Therefore, it is essential that ^13^C-MFA software supports the flexible composition of analysis workflows to appropriately address the research question at hand while also being streamlined for computational efficiency.

The simulation step is the foundational centerpiece of any evaluation workflow, because it generates isotope labeling data given a metabolic model including atom transitions, parameter values, and a measurement configuration. Reliable and fast simulation of the labeling data enables addressing novel types of questions, such as the impact of model uncertainty on the estimated fluxes (Theorell *et al.* 2024). Several ^13^C-MFA software packages are available (Section 4, available as supplementary data at *Bioinformatics* online for a selection). These use different flux coordinate systems and state-space representations, such as the prominent cumomers or elementary metabolite units (EMU), which determine the properties of the underlying mathematical equations. However, none of these tools cover the full range of now-possible applications, including isotopic stationary and nonstationary ^13^C-MFA variants with various measurement configurations and multi-isotopic tracers, nor do they select the numerically most beneficial state-space representation. Also, these tools rarely offer the flexibility to accommodate new fitting, statistical, or experimental design approaches.

The open high-performance simulator 13CFLUX(v3) supports the full range of now possible ^13^C-MFA scenarios, including INST. Its universality is based on the ability to simulate any desired labeling state of any metabolite within a given model for any input labeling and at any point in time (including t=∞). 13CFLUX(v3) builds on the universal flux modeling language FluxML (Beyß *et al.* 2019), and improves upon the performance of its predecessor 13CFLUX2 (Weitzel *et al.* 2013), extending it to INST. Its ground-up new software architecture enables extensible and scalable analyses, facilitating workflow automation, empowering researchers to tackle complex biological questions and applications.

## 2 Approach and implementation

### 2.1 Software architecture

**Figure 1.**
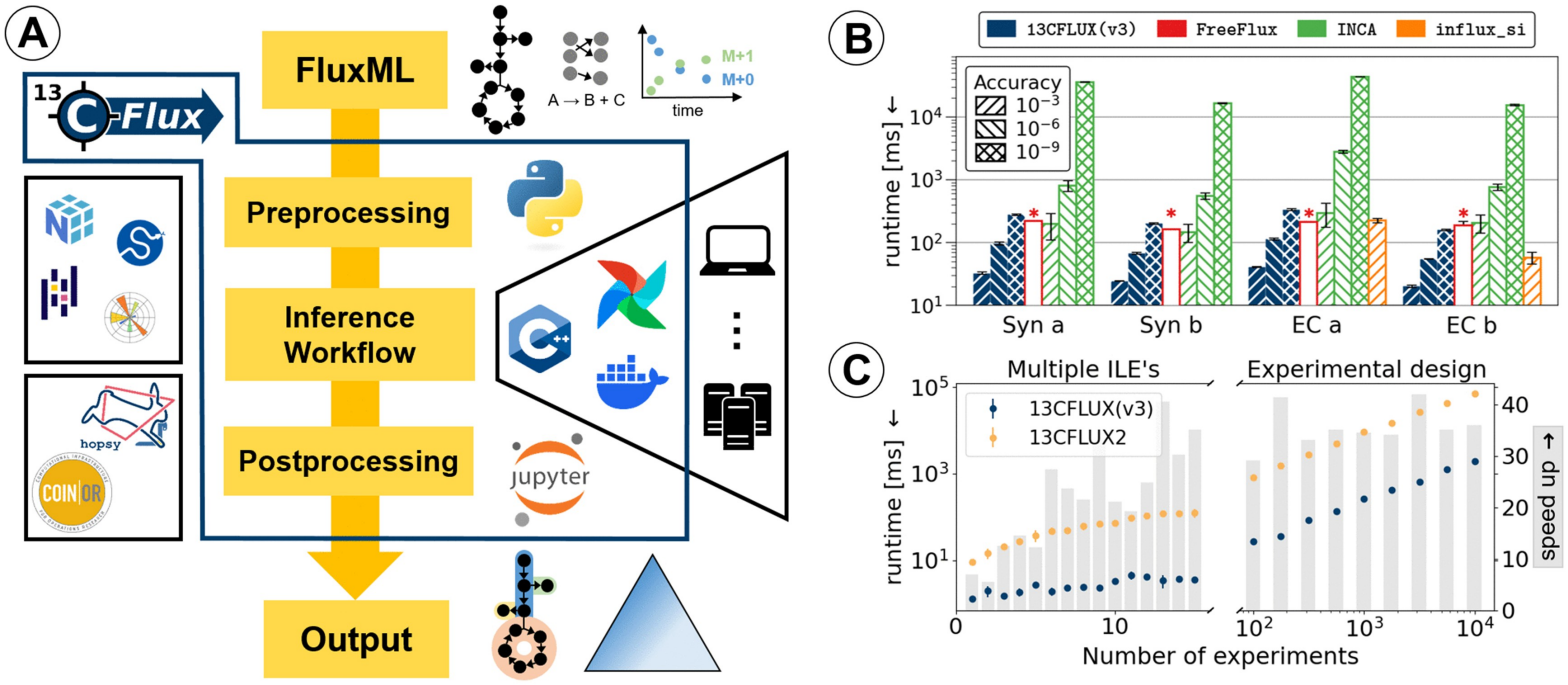
(A) 13CFLUX(v3) supports flexible, scalable workflows through a high-level Python API that interfaces with the high-performance C++ simulation backend; containerization ensures portability. (B) Performance comparison (single-core) of INST simulation wall clock time for low-, mid-, and high-accuracy calculations (*: numerical accuracy uncontrollable, see Section 5.2, available as supplementary data at *Bioinformatics* online). Mean and standard deviation over 100 repetitions. (C) Speed-up of 13CFLUX(v3) versus 13CFLUX2 for simulating multiple IST ILEs.

The 13CFLUX(v3) architecture integrates a C++ simulation backend with a Python frontend for performance and to conveniently leverage third-party Python libraries like NumPy, SciPy, or Matplotlib (Fig. 1A). This cross-language approach, realized using pybind11 (ver. 3.0.1), compiles the backend and Python bindings into shared libraries accessible to all actively supported Python interpreters (vers. 3.9–13). Advanced exception handling ensures that error and warning messages are passed from the C++ backend to Python.

Loading a FluxML file in Python creates a simulator object consisting of the dimension-reduced underlying isotope labeling system and data structures tailored to the given ^13^C-MFA model. This object provides access to simulated labeling data, parameter sensitivities, residuals (variance-weighted difference between simulated and measured data), and gradients, aiding system analysis and flux estimation.

Compared to 13CFLUX2, the C++ code has been fully refactored, e.g. by replacing custom matrix/vector operations with those provided by Eigen (ver. 3.4), thereby reducing the lines of code (LOC) from over 130 000 to <15 000 (Section 3.1, available as supplementary data at *Bioinformatics* online). This, along with unit testing, enhances maintainability and software quality. The code is written in C++17 (ISO/IEC 14882) and compiled with standard tools like GCC or Clang to highly optimized machine code. CMake (vers. 3.15–4.1) manages compilation and testing. 13CFLUX(v3) is deployable as a Python package/wheel e.g. from the Python Package Index (x3cflux) or as a Docker container, providing a ready-to-use environment.

### 2.2 C++ simulation backend

Battle-proven algorithms are key for achieving simulation performance with high degree of application universality. 13CFLUX(v3) features two universal state-space representations of isotopic labeling, namely cumomers (Wiechert 2001) and EMUs (Antoniewicz *et al.* 2007). For a FluxML model, a topological graph analysis and decomposition of the cumomer/EMU isotope labeling balance equations produces dimension-reduced state-spaces [i.e. essential cumomers or EMUs (Weitzel *et al.* 2007)]. A heuristic maximizes the reduction by automatically deciding the formulation (Section 2.4.1, available as supplementary data at *Bioinformatics* online). The dimension-reduced labeling systems take the form of nonlinearly coupled “cascaded” systems (Section 1.1, available as supplementary data at *Bioinformatics* online), which, depending on the data type, reduce to either algebraic equation systems (AE; IST) or ordinary differential equation systems (ODE; INST). Typical system sizes exceed 1000 dimensions (Section 1.2, available as supplementary data at *Bioinformatics* online).

Taking advantage of the systems’ sparsity, the AEs are solved using sparse LU factorization via Gaussian elimination with the *SparseLU* algorithm from Eigen. The SUNDIALS suite (ver. 6.6) is used to solve the INST ODEs. In particular, we use a customized version of *CVODE*, a A(α)- and L(α)-stable multistep Backward Differentiation Formula (BDF) method with step size and order-control, which is suitable for (non)stiff ODE integration (*L*-stability is beneficial as it is robust for integrating stiff ODEs even for large step sizes). The linear system characteristics of the AEs underlying the BDF schemes is leveraged through replacing the iterative *GMRES* algorithm by a 1-step *SparseLU* factorization (Section 2.1, available as supplementary data at *Bioinformatics* online). Besides *CVODE*, an *L*-stable single-step singly diagonally implicit Runge-Kutta method is implemented. All ODE integrators implement adaptive step size control (Section 2.2, available as supplementary data at *Bioinformatics* online), required in all settings where parameter values or design variables vary unpredictably, such as flux estimation or experimental design. Numerical accuracy is validated by comparison with analytical and reference solutions (Section 2.3, available as supplementary data at *Bioinformatics* online). The AE/ODE labeling systems and their analytically derived sensitivity systems are solved with the same solvers, enhanced by multi-threaded shared memory parallelization via OpenMP(vers. 3.0+), which exploits the independence of the sensitivity systems. Labeling states for sets of isotopically labeled substrates, under the same or different measurement configurations, are computed through low-level optimization of the associated AE/ODE systems (Section 1.3, available as supplementary data at *Bioinformatics* online).

### 2.3 Flexible ^13^C-MFA workflows

A key goal in developing 13CFLUX(v3) was to enable reproducible (automatable, portable), scalable, and transparent workflows without requiring extensive coding expertise. To this end, we adopted an object-oriented approach that abstracts the internal state-space representation and encapsulates the dimension-reduced model in a polymorphic simulator object (Section 2.1). This allows users to specify model parameters (fluxes, pool sizes) and simulation variables (solvers, accuracy, etc.), and to configure tasks like multi-start parameter fitting with minimal code via a high-level Python API. For example, multi-start fitting requires a single LOC and switching to a third-party optimizer involves only two changes (Section 3.2, available as supplementary data at *Bioinformatics* online). This design balances ease of use with flexibility, supporting the integration of external algorithms and enabling the setup of automated production workflows, e.g. via powerful workflow orchestration platforms such as Apache Airflow (https://airflow.apache.org/, Section 7, available as supplementary data at *Bioinformatics* online).

Documentation and Jupyter notebooks provide templates for both standard and advanced workflows. 13CFLUX(v3) also issues expressive error messages and warnings at semantic, syntactic, logic, and numeric levels (Section 3.3, available as supplementary data at *Bioinformatics* online). Portability is supported through Docker containerization, which decouples workflow development from compute resources. This facilitates reproducible and scalable execution, from laptops to high-performance clusters.

## 3 Results

We demonstrate the utility of 13CFLUX(v3) via benchmarks against state-of-the-art (SOTA) simulators and, for the first time, Bayesian uncertainty quantification in INST ^13^C-MFA.

### 3.1 Performance benchmark

Benchmarking ^13^C-MFA simulations includes consideration of the model, measurement configuration (affecting the degree of dimension reduction), state-space representation (Section 2.2), and solver accuracy (specific to INST). We compared the simulation wall clock times of 13CFLUX(v3) with three SOTA simulators (FreeFlux, INCA, influx_si) for two organisms using published models [*E. coli* (EC) (Young 2014), *Synechocystis* sp. PCC6803 (Syn) (Wu *et al.* 2023)], two measurement configurations (a, b), and different accuracies (see Section 8, available as supplementary data at *Bioinformatics* online for details of the models). Figure 1B and the results in Section 5, available as supplementary data at *Bioinformatics* online, show that 13CFLUX(v3) outperforms the other tools in all categories by far. In addition, 13CFLUX(v3) facilitates scalable simulations of multiple data and parameter sets, achieving runtimes that are ∼40 times faster than those of 13CFLUX2 (Fig. 1C). This factor extends to Jacobian computations and significantly speeds up the evaluation of tracer designs (Section 2.4.2, available as supplementary data at *Bioinformatics* online).

### 3.2 Unlocking Bayesian INST ^13^C-MFA

Bayesian approaches have recently complemented the statistical toolkit for classical ^13^C-MFA (Theorell *et al.* 2017, Backman *et al.* 2023, Hogg *et al.* 2023), but long simulation times have hindered their application to Bayesian INST ^13^C-MFA so far. Leveraging the performance of 13CFLUX(v3) together with efficient MCMC algorithms for linearly constrained problems, we demonstrate, to our knowledge, the first Bayesian inference for INST ^13^C-MFA. We implemented a Python workflow that uses 13CFLUX(v3) as the simulation engine and uses the specialized library hopsy for MCMC sampling (Paul *et al.* 2024). Due to its high-level Python API, integration of the two packages requires only 20 LOCs, with the full analysis workflow implemented under 300 LOCs (Section 6.1, available as supplementary data at *Bioinformatics* online). The workflow was containerized with Docker and executed on an HPC cluster. Exemplary posterior probability distributions along with further analysis details are provided in Section 6.2, available as supplementary data at *Bioinformatics* online.

## 4 Conclusion

The new simulation engine 13CFLUX(v3) handles the full spectrum of ^13^C-MFA scenarios, spanning IST and INST analyses, multi-experiment setups, multi-isotope tracers and complex measurement configurations. Its C++ simulation core ensures efficiency and reliability, while the Python API enables seamless interaction with advanced data analysis tools, workflow customization and automation, thereby supporting both current and future research needs. By uniting performance, reliability, flexibility and openness, 13CFLUX(v3) establishes a sustainable platform for ^13^C-MFA, empowering researchers to study complex biological systems and integrate comprehensive datasets to gain quantitative insight into metabolic processes.

## Supplementary Material

btaf630_Supplementary_Data
