# Supplementary material for: 13CFLUX - third-generation high-performance engine for isotopically (non)stationary 13C metabolic flux analysis
Source: Bioinformatics. 2025 Nov 18;41(12):btaf630. doi: 10.1093/bioinformatics/btaf630 (PMC12696647; doi:10.1093/bioinformatics/btaf630)
Supplement: btaf630_Supplementary_Data [file btaf630_supplementary_data.zip › Stratmann_13CFLUX_BIOINF_R.1 - Supplementary Information.pdf]

Supplementary Information for

13CFLUX – Third-generation high-performance engine for isotopically (non)stationary ^{13}C metabolic flux analysis

Anton Stratmann [1,2], Martin Beyß [1,2], Johann F. Jadebeck [1,2], Wolfgang Wiechert [1,2] and Katharina Nöh [1,*]

[1] Institute of Bio- and Geosciences, IBG-1: Biotechnology, Forschungszentrum Jülich, Wilhelm-Johnen-Str., 52428 Jülich, Germany

[2] Computational Systems Biotechnology (AVT.CSB), RWTH Aachen University, Forckenbeckstr., 52074 Aachen, Germany

Corresponding author: k.noeh@fz-juelich.de

Contents

S.1 Isotope labeling systems **3**
S.1.1 Cascaded isotope labeling and parameter sensitivity equations . . . 3
S.1.2 Dimensionality of labeling systems . . . 3
S.1.3 Efficient labeling state-space formulation for multiple labeling datasets . . . 4

S.2 Numerical labeling simulation with `13CFLUX(v3)` **6**
S.2.1 Taylored differential equation solvers . . . 6
S.2.2 Adaptive step-size control . . . 6
S.2.3 Numerical solution quality . . . 6
S.2.3.1 Numerical accuracy for an analytically tractable test system . . . 6
S.2.3.2 Numerical reliability of INST simulations . . . 8
S.2.3.3 Consistency of IST and INST numerical solutions . . . 8
S.2.4 Scalability of `13CFLUX(v3)` . . . 9
S.2.4.1 Simulation of cumomer- and EMU-based labeling systems in `13CFLUX(v3)` . . . 9
S.2.4.2 Efficient simulation of datasets from multiple labeling experiments . . . 11

S.3 `13CFLUX(v3)` implementation details **13**
S.3.1 Software statistics . . . 13
S.3.2 Interoperation of `13CFLUX(v3)` with third-party libraries . . . 13
S.3.3 Expressive error messages . . . 14

S.4 Simulator collection **15**

S.5 Simulator comparison **16**
S.5.1 Comparison of simulated labeling states . . . 16
S.5.2 Performance benchmark . . . 17
S.5.3 Parameter variation . . . 17
S.5.4 Scalability of INST simulations . . . 18

S.6 Novel application: Bayesian INST ^{13}C-MFA **20**
S.6.1 Efficient MCMC sampling using `13CFLUX(v3)` . . . 20
S.6.2 Application . . . 21

S.7 `Airflow`'ing `13CFLUX(v3)` production workflows **23**

S.8 Metabolic models **26**
S.8.1 Linear pathway model 26
S.8.2 *Escherichia coli* (EC) 27
S.8.3 *Synechocystis* (Syn) 28

Availability and Reproducibility:
Model files and computational scripts are available at https://github.com/JuBiotech/Supplement-to-Stratmann-et-al.-Bioinformatics-2025. A Docker container is provided that allows for the reproduction of the SI figures. A snapshot of the software that is used to perform the experiments presented in this work is archived on Zenodo https://doi.org/10.5281/zenodo.17476359. Documentation and examples are availanle at https://x3cflux.readthedocs.io/.

S.1 Isotope labeling systems

S.1.1 Cascaded isotope labeling and parameter sensitivity equations

The fractional labeling enrichment in ^{13}C-MFA is described by so-called cascaded labeling differential or algebraic equation systems (Nöh et al., 2006). In the dynamic case, the cascaded systems consist of a sequence of K lower-dimensional linear initial value problems (IVP) for the labeling states ${}^{k}\mathbf{x}, k = 1(1)K$ (cumomer or EMU). Given the parameters, i.e., fluxes $\mathbf{v}$ and pool sizes $\mathbf{X}$ (if applicable), as well as the substrate label composition ${}^{\text{inp}}\mathbf{x}$, the IVPs are given by

$$\text{diag}({}^{k}\mathbf{X}) \cdot {}^{k}\dot{\mathbf{x}} = {}^{k}\mathbf{A}(\mathbf{v}) \cdot {}^{k}\mathbf{x} + {}^{k}\mathbf{b}(\mathbf{v}, {}^{0}\mathbf{x}, \ldots, {}^{k-1}\mathbf{x}, {}^{\text{inp}}\mathbf{x}), \quad k = 1(1)K \tag{S1}$$

with initial labeling states ${}^{k}\mathbf{x}(t = 0) = {}^{k}\mathbf{x}_0, k = 1(1)K$. For $t \to \infty$, ${}^{k}\mathbf{x}$ asymptotically approach constant values and the cascaded IVP simplifies to an algebraic labeling system

$$\mathbf{0} = {}^{k}\mathbf{A}(\mathbf{v}) \cdot {}^{k}\mathbf{x} + {}^{k}\mathbf{b}(\mathbf{v}, {}^{0}\mathbf{x}, \ldots, {}^{k-1}\mathbf{x}, {}^{\text{inp}}\mathbf{x}) \quad k = 1(1)K \tag{S2}$$

The ^{13}C-MFA variants given by Eqs. (S1) and (S2) are termed isotopically nonstationary (INST) and isotopically stationary (IST) ^{13}C-MFA, respectively. Notably, the state space representation (cumomer or EMU) affects the dimensions of the cascaded labeling systems: while ${}^{k}\mathbf{A}$ and $\text{diag}({}^{k}\mathbf{X})$ are matrices, the inhomogeneous term, ${}^{k}\mathbf{b}$, and the labeling states, ${}^{k}\mathbf{x}$, are vector-valued for cumomers, but matrix-valued for EMUs (Wiechert and Wurzel, 2001, Young et al., 2008).

When the parameter values in the Eqns. (S1) and (S2) are unknown, as in the case of parameter estimation, determining the sensitivity of their solution with respect to parameter value perturbations is an important step. Sensitivities in Eq. (S1) with respect to fluxes and pool sizes are determined by solving the cascaded sensitivity IVPs

$$\begin{aligned}
\text{diag}({}^{k}\mathbf{X}) \cdot (\dot{\partial_{\mathbf{v}}{}^{k}\mathbf{x}}) &= {}^{k}\mathbf{A} \cdot \partial_{\mathbf{v}}{}^{k}\mathbf{x} + \partial_{\mathbf{v}}{}^{k}\mathbf{A} \cdot {}^{k}\mathbf{x} + \partial_{\mathbf{v}}{}^{k}\mathbf{b} + \sum_{i=0}^{k-1} \partial_{{}^{i}\mathbf{x}}{}^{k}\mathbf{b} \cdot \partial_{\mathbf{v}}{}^{i}\mathbf{x}, \qquad (\partial_{\mathbf{v}}{}^{k}\mathbf{x})(t = 0) = \mathbf{0} \\
\text{diag}({}^{k}\mathbf{X}) \cdot (\dot{\partial_{\mathbf{X}}{}^{k}\mathbf{x}}) &= {}^{k}\mathbf{A} \cdot \partial_{\mathbf{X}}{}^{k}\mathbf{x} - \partial_{\mathbf{X}}\text{diag}({}^{k}\mathbf{X}) \cdot {}^{k}\dot{\mathbf{x}} + \sum_{i=0}^{k-1} \partial_{{}^{i}\mathbf{x}}{}^{k}\mathbf{b} \cdot \partial_{\mathbf{X}}{}^{i}\mathbf{x}, \qquad (\partial_{\mathbf{X}}{}^{k}\mathbf{x})(t = 0) = \mathbf{0}
\end{aligned} \tag{S3}$$

for $k = 1(1)K$, where $\partial_{.}$ is a shortcut for $\mathrm{d}/\mathrm{d}_{.}$, and the dependencies of ${}^{k}\mathbf{A}$ and ${}^{k}\mathbf{b}$ are omitted for notational brevity. For the IST case, which does not depend on the pool sizes, the sensitivity system reduces to

$$\mathbf{0} = {}^{k}\mathbf{A} \cdot \partial_{\mathbf{v}}{}^{k}\mathbf{x} + \partial_{\mathbf{v}}{}^{k}\mathbf{A} \cdot {}^{k}\mathbf{x} + \partial_{\mathbf{v}}{}^{k}\mathbf{b} + \sum_{i=0}^{k-1} \partial_{{}^{i}\mathbf{x}}{}^{k}\mathbf{b} \cdot \partial_{\mathbf{v}}{}^{i}\mathbf{x} \tag{S4}$$

S.1.2 Dimensionality of labeling systems

The dimension of the cumomer and EMU labeling systems increases with the size of the network model (Weitzel et al., 2007). Analyzing the topological structure of cascaded labeling systems using graph-theoretic concepts significantly reduces the dimension of the systems, yielding essential cumomer and EMU state-space representations (Weitzel et al., 2007, Wiechert et al., 2013). Essential cumomer and EMU state-space formulations follow the notion of tracing observed labeling fragments backward to the sources of the label input (Antoniewicz et al., 2007). Consequently, essential cumomer or EMU state spaces emerge with different dimensionalities and forms (see SI Table S.1). These, in turn, affect the speed of the computational solutions for labeling and parameter sensitivity systems (see SI Section S.1.1). For this reason, `13CFLUX(v3)` supports both state-space representations and uses an automated selection heuristic to select the most efficient one.

		MS		MSMS	
	Config.	Cumomer	EMU	Cumomer	EMU
EC_	b	151×1, 46×1	149×2, 46×3	151×1, 46×1	151×2, 46×3
	#8	152×1,<br>184×1,120×1,<br>51×1, 15×1, 2×1	149×2, 90×3,<br>48×4, 11×5, 5×6,<br>2×7	152×1, 184×1,<br>120×1, 51×1,<br>15×1, 2×1	152×2, 184×3,<br>120×4, 51×5,<br>15×6, 2×7
	#20	176×1, 268×1,<br>231×1, 144×1,<br>74×1, 30×1, 8×1,<br>1×1	150×2, 90×3,<br>49×4, 20×5, 8×6,<br>2×7, – , 1×9	179×1, 288×1,<br>282×1, 221×1,<br>154×1, 88×1,<br>36×1, 9×1, 1×1	179×2, 288×3,<br>282×4, 221×5,<br>154×6, 88×7,<br>36×8, 9×9, 1×10
	a	184×1, 289×1,<br>272×1, 206×1,<br>145×1, 86×1,<br>36×1, 9×1, 1×1	150×2, 92×3,<br>49×4, 22×5, 9×6,<br>2×7, – , 1×9, 1×10	184×1, 289×1,<br>272×1, 206×1,<br>145×1, 86×1,<br>36×1, 9×1, 1×1	184×2, 289×3,<br>272×4, 206×5,<br>145×6, 86×7,<br>36×8, 9×9, 1×10
	full	247×1, 536×1,<br>782×1, 886×1,<br>837×1, 656×1,<br>404×1, 183×1,<br>57×1, 11×1, 1×1	247×2, 536×3,<br>782×4, 886×5,<br>837×6, 656×7,<br>404×8, 183×9,<br>57×10, 11×11,<br>1×12	247×1, 536×1,<br>782×1, 886×1,<br>837×1, 656×1,<br>404×1, 183×1,<br>57×1, 11×1, 1×1	247×2, 536×3,<br>782×4, 886×5,<br>837×6, 656×7,<br>404×8, 183×9,<br>57×10, 11×11,<br>1×12
Syn_	b	129×1, 138×1,<br>46×1	129×2, 92×3, 46×3		
	a	129×1, 232×1,<br>252×1, 176×1,<br>77×1, 19×1, 2×1	129×2, 92×3,<br>46×4, 19×5, 14×6,<br>5×7, 2×8		
	full	129×1, 232×1,<br>252×1, 176×1,<br>77×1, 19×1, 2×1	129×2, 232×3,<br>252×4, 176×5,<br>77×6, 19×7, 2×8		

Table S.1: **State-space dimensionalities of typical ^{13}C-MFA models.** For two exemplary models EC and Syn (see SI Section S.2.4.1), dimensions of essential cumomer and EMU system matrices are listed, resolved by cascade levels. Notice that the dimensionality depends heavily on the measurement configuration (second column) accompanying the ^{13}C-MFA models. #8 and #20 give two randomly selected measurement configurations, full the unreduced state-space representation.

S.1.3 Efficient labeling state-space formulation for multiple labeling datasets

Using the same ^{13}C-MFA-model to evaluate multiple isotope labeling datasets that are acquired under very similar conditions has become mainstream (Long and Antoniewicz, 2019). This requires solving one cascaded system, either in the form of Eq. (S1) or Eq. (S2), per dataset. We showcase a computationally advantageous way to deal with the evaluation of multiple datasets, with the IST ^{13}C-MFA variant at hand (the derivation for the INST case is analogous).

Given N labeling datasets, the cascaded IST equation system are written as

$$\mathbf{0} = \begin{pmatrix} {}^{k}\mathbf{A}(\mathbf{v}) & & \\ & \ddots & \\ & & {}^{k}\mathbf{A}(\mathbf{v}) \end{pmatrix} \cdot \begin{pmatrix} {}^{k}\mathbf{x}_1 \\ \vdots \\ {}^{k}\mathbf{x}_N \end{pmatrix} + \begin{pmatrix} {}^{k}\mathbf{b}(\mathbf{v}, {}^{0}\mathbf{x}_1, \ldots, {}^{k-1}\mathbf{x}_1, {}^{\mathrm{inp}}\mathbf{x}_1) \\ \vdots \\ {}^{k}\mathbf{b}(\mathbf{v}, {}^{0}\mathbf{x}_N, \ldots, {}^{k-1}\mathbf{x}_N, {}^{\mathrm{inp}}\mathbf{x}_N) \end{pmatrix}, \; k = 1(1)K \qquad \text{(S5)}$$

The most costly evaluation in solving Eq. (S5) is in the inversion of the block-diagonal matrix. Recognizing that the matrices on the diagonal are the same for each dataset, instead of solving the complete

system Eq. (S5), `13CFLUX(v3)` solves the augmented labeling system

$$\mathbf{0} = {}^k\mathbf{A}(\mathbf{v}) \cdot {}^k\mathbf{z} + {}^k b(\mathbf{v}, {}^0\mathbf{z}, \ldots, {}^{k-1}\mathbf{z}, {}^{\mathrm{inp}}\mathbf{z}), \quad k = 1(1)K \tag{S6}$$

for the extended labeling states ${}^k\mathbf{z} = \left({}^k\mathbf{x}_1, \ldots, {}^k\mathbf{x}_N\right)$. Thereby, the inversion of ${}^k\mathbf{A}(\mathbf{v})$ has only to be performed once, which reduces computations by $N-1$ fold. Clearly, this reformulation works for cumomers and EMU state-space systems. The resulting speed-up factors are shown in Figure 1C in the main text. Notably, this derivation also applies to cumomer- and EMU-based parameter sensitivity systems in Eqns. (S4) and (S3), where it accelerates experimental design calculations (see SI Section S.2.4.2).

S.2 Numerical labeling simulation with `13CFLUX(v3)`

S.2.1 Taylored differential equation solvers

In `13CFLUX(v3)`, the IVP systems in Eq. (S1) and Eq. (S3) are solved numerically. For this, a single and multi-step methods are implemented; in particular, a Singly-Diagonally Implicit Runge-Kutta (SDIRK) method of order 4 (Hairer and Wanner, 1996), and Backward Differentiation Formula (BDF) of order 1-5 (Hindmarsh et al., 2005) are available. Both SDIRK and BDF solvers require the solution of algebraic equation systems for each cascade level $k = 1(1)K$, and for every time step of the integration window. Essentially, per step this amounts to the solution of an algebraic equation of the general form

$$\left(\mathbf{I} + \lambda \cdot \mathrm{diag}({}^{k}\mathbf{X})^{-1} \cdot {}^{k}\mathbf{A}(\mathbf{v})\right) \cdot \hat{\mathbf{y}} = \mathbf{z} \tag{S7}$$

where λ and $\mathbf{z}$ are solver-specific, and $\mathbf{I}$ is the identity matrix. The original implementation of the CVODE solver (Hindmarsh et al., 2005) employs an iterative scheme to solve Eq. (S7). The BDF implementation in `13CFLUX(v3)`, however, solves the system in one step using lower-upper (LU) factorization, which exploits the system structure of the cascaded labeling systems and the sparsity of the matrices ${}^{k}\mathbf{A}$, thereby reducing the numerical complexity. The same feature is utilized when solving the sensitivity IVPs in Eq. (S3).

S.2.2 Adaptive step-size control

State-of-the-art IVP solvers are equipped with adaptive step-size control, which tries to maintain the accuracy of numerical IVP solutions within a certain tolerance (Hairer and Wanner, 1996). The adaptive step-size control is based on an estimate of the local numerical (discretization) error, which is determined in each integration step. According to this estimate, the integration step-size is set so that the local error does not exceed a user-specified error tolerance. If this local error exceeds the demanded tolerance level, a smaller step-size is automatically selected. By construction, this also prevents the overall global numerical error of the IVP solution from increasing too much. Therefore, adaptive step-size control is essential to ensure that the numerical solution process is both reliable and efficient.

SDIRK and BDF schemes implemented in `13CFLUX(v3)` are equipped with adaptive step-size control, with the option of adjusting the relative and absolute integration tolerances, tol_{rel} and tol_{abs}, respectively. The relative tolerance relates to the error relative to the IVP solution, whereas the absolute tolerance gives the absolute limit, which protects the solution against round-off errors. The cumulative contributions of tol_{rel} and tol_{abs} define the numerical tolerance that is used for estimating the integration step-size for the next time-step. For SDIRK, the step-size control relies on error estimation using an embedded scheme (Hairer and Wanner, 1996). For BDF, the interpolation technique of Nordsieck is utilized (Nordsieck, 1962).

Because the final numerical solution error accumulates over the integration steps, it is important to choose tolerance levels wisely. A conservative rule of thumb is to choose tolerances that are two orders of magnitude smaller than the acceptable error. For a common INST ^{13}C-MFA simulation, a relative tolerance of $tol_{rel} = 10^{-6}$ and an absolute tolerance of $tol_{abs} = 10^{-9}$ mean that the IVP solution is about two orders of magnitude more accurate than the measurements $\mathcal{O}(10^{-4})$. These tolerance values are the default choice in `13CFLUX(v3)`.

S.2.3 Numerical solution quality

S.2.3.1 Numerical accuracy for an analytically tractable test system

To test correctness of the IVP solver implementations in `13CFLUX(v3)`, as it is common practice in the field, we test the accuracy of the numerical solution with a simple test equation

$$\begin{pmatrix} A & 0 \\ 0 & 1 \end{pmatrix} \cdot \frac{d}{dt}\begin{pmatrix} a \\ b \end{pmatrix} = \begin{pmatrix} -(1+\tau) & \tau \\ 1+\tau & -1 \end{pmatrix} \cdot \begin{pmatrix} a \\ b \end{pmatrix} + \begin{pmatrix} 1 \\ 0 \end{pmatrix} \tag{S8}$$

Despite its simplicity, this system exhibits the dynamic characteristics of isotope labeling systems: Increasing the value of τ and decreasing the value of A worsens the condition of the system, making it increasingly stiff, and therefore more difficult to solve numerically. This is a well-known challenge for IVP solvers (Dahlquist, 1963).

The exact solution of Eq. (S8) is given by

$$
\begin{aligned}
a_{\text{exact}}(t) = & \frac{1}{(2\tau^2-2)\sqrt{A^2+(4\,\tau^2+2\,\tau-2)\,A+(1+\tau)^2}} \\
& \cdot\Bigg(\left(2\,\tau^2+A+\tau+\sqrt{A^2+(4\,\tau^2+2\,\tau-2)\,A+(1+\tau)^2}-1\right) \\
& \quad \cdot \mathrm{e}^{-\frac{A+\tau+1-\sqrt{A^2+(4\,\tau^2+2\,\tau-2)A+(1+\tau)^2}}{2A}t} \\
& + \left(-2\,\tau^2-A-\tau+\sqrt{A^2+(4\,\tau^2+2\,\tau-2)\,A+(1+\tau)^2}+1\right) \\
& \quad \cdot \mathrm{e}^{-\frac{A+\tau+1+\sqrt{A^2+(4\,\tau^2+2\,\tau-2)A+(1+\tau)^2}t}{2A}t} \\
& - 2\sqrt{A^2+(4\,\tau^2+2\,\tau-2)\,A+(1+\tau)^2}\Bigg)
\end{aligned}
\tag{S9}
$$

$$
\begin{aligned}
b_{\text{exact}}(t) = & \frac{1}{(2\tau-2)\sqrt{A^2+(4\,\tau^2+2\,\tau-2)\,A+(1+\tau)^2}} \\
& \cdot\Bigg(\left(A+\tau+\sqrt{A^2+(4\,\tau^2+2\,\tau-2)\,A+(1+\tau)^2}+1\right) \\
& \quad \cdot \mathrm{e}^{-\frac{A+\tau+1-\sqrt{A^2+(4\,\tau^2+2\,\tau-2)A+(1+\tau)^2}}{2A}t} \\
& + \left(-A-\tau+\sqrt{A^2+(4\,\tau^2+2\,\tau-2)\,A+(1+\tau)^2}-1\right) \\
& \quad \cdot \mathrm{e}^{-\frac{A+\tau+1+\sqrt{A^2+(4\,\tau^2+2\,\tau-2)A+(1+\tau)^2}t}{2A}t} \\
& - 2\sqrt{A^2+(4\,\tau^2+2\,\tau-2)\,A+(1+\tau)^2}\Bigg)
\end{aligned}
\tag{S10}
$$

Knowing the exact solution allows defining the global numerical error of the numerical solution taken at N time steps t_i within the integration window $[0, T]$. Precisely, we define the global numerical error as follows

$$
e_{num,exact} = \max_{1\le i\le N} \|\mathbf{x}_{num}(t_i) - \mathbf{x}_{exact}(t_i)\|_2,\ t_i \in [0, T] \tag{S11}
$$

Eq. (S11) represents the worst-case deviation of the calculated numerical solution from the analytical one across the entire integration steps. We use this error metric here to analyze the accuracy of the numerical IVP solvers that have been implemented in `13CFLUX(v3)`.

With the test IVPs in Eq. (S8) at hand, different parameter combinations of $\tau \in [10, 1000]$ and $A \in [1/10, 1/1000]$ are chosen to create test systems with different stiffnesses, as indicated by condition numbers ranging from low $(2 \cdot 10^2)$ to high $(2 \cdot 10^6)$ values. The IVPs are solved within the integration domain $[0, 100]$ with settings for low, medium, and high relative tolerances, i.e., $tol_{rel} = 10^{-3}, 10^{-6}$, and 10^{-9}, respectively. In all cases, the absolute tolerance tol_{abs} is set to $tol_{rel} \cdot 10^{-3}$ to prevent near-zero values from escaping the error control.

Figure S.1 shows the global numerical errors $e_{num,exact}$ for the SDIRK and BDF solvers applied to the set of test IVPs. The achieved numerical accuracy is within one order of magnitude of the configured relative tolerance. Thus, both solvers show reliable control over the global numerical error and do not produce overly precise results, meaning that computational resources are not wasted by performing more time steps than necessary. However, for the test IVPs, we find that the step-size control of the SDIRK solver is more conservative than that of the BDF schemes. Abstracting from these results, we recommend selecting a relative tolerance of 10^{-6} for the BDF solver to achieve $\mathcal{O}(10^{-5})$ solution accuracy (see also SI Section S.2.3.2).

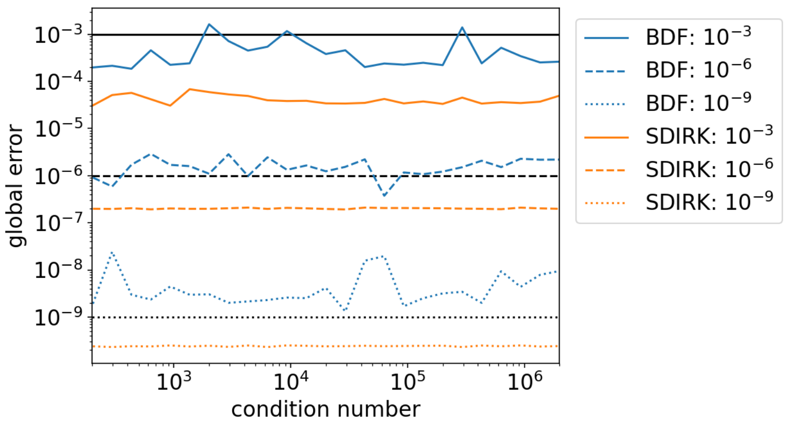

Figure S.1: **Global numerical errors for test IVPs with differing degrees of ill-conditionedness**. The BDF and SDIRK solvers implemented in `13CFLUX(v3)` maintain a global numerical error $e_{num,exact}$ close to the local tolerance, regardless of the condition number of the test IVP. The absolute tolerances are set to $tol_{rel} \cdot 10^{-3}$ in all cases.

S.2.3.2 Numerical reliability of INST simulations

For real-world ^{13}C-MFA models, the exact solution is no longer analytically tractable. To assess the global numerical error for real ^{13}C-MFA models, we therefore resort to a highly accurate SDIRK solution computed with a very small tolerance ($tol_{rel} = 10^{-12}$, $tol_{abs} = 10^{-15}$), which we use as reference IVP solution $\mathbf{x}_{ref}$. The degree of ill-conditionedness of the IVPs affect the accuracy of the numerical solutions. Therefore we randomly select 10 feasible parameter constellations and approximate the global numerical error $e_{num,ref}$ with respect to the reference solution according to

$$e_{num,ref} = \max_{1 \le i \le N} \|\mathbf{x}_{num}(t_i) - \mathbf{x}_{ref}(t_i)\|_2, \ t_i \in [0, T] \tag{S12}$$

For the *Synechocystis* model Syn_a (see SI Section S.8.3 for details), Figure S.2 shows the mean and standard deviation of the approximated global numerical error for SDIRK and BDF solvers. We observe that the approximated global numerical error $e_{num,ref}$ is linearly correlated (in log-log space) with the relative tolerance tol_{rel}. The desired tolerances are always met within one order of magnitude. Hence, we conclude that both numerical IVP solvers provide reliable and robust solutions to real-world ^{13}C-MFA models. In particular, users of `13CFLUX(v3)` have the ability to provide effective error control over the implemented numerical IVP solvers to meet individually preferences.

S.2.3.3 Consistency of IST and INST numerical solutions

So far, we have evaluated the numerical accuracy of our INST solvers with different tolerances and realistic networks by comparing them to a ultrahigh-accuracy reference solution generated using the SDIRK solver implemented in `13CFLUX(v3)`. Because there is no provably correct alternative simulator is available for independently verifying the numerical solution, we perform an additional consistency check utilizing the characteristics of the isotope labeling systems. Specifically, we verify that the labeling states of the INST IVPs in Eq. (S1) converge to the solution of the IST equations in Eq. (S2) for very large t (Wiechert and Wurzel, 2001).

Exploiting this fact allows us to study the consistency of the numerical IST and INST solutions, given a single FluxML model file. We here take the *E. coli* EC_a model and calculate (i) the IST solution ($\mathbf{x}(\infty)$) and (ii) the labeling state at $t = 10{,}000{,}000$ s ($\mathbf{x}(10{,}000{,}000)$) for 1,000 random pool size parameter configurations. For each of these configurations, we determine the maximal absolute difference between the simulated IST and INST solutions in terms of the observed labeling patterns as follows

$$e_{IST,INST} = \|\mathbf{x}(\infty) - \mathbf{x}(10{,}000{,}000)\|_\infty \tag{S13}$$

Figure S.3 shows that the INST solution at a large t generated using the BDF solver with $tol_{rel} = 10^{-6}$ and $tol_{abs} = 10^{-9}$ matches the IST solution with a maximum deviation of $1.2 \cdot 10^{-7}$, which is one order of magnitude lower than the solvers' relative tolerance. We therefore conclude that the numerical solution

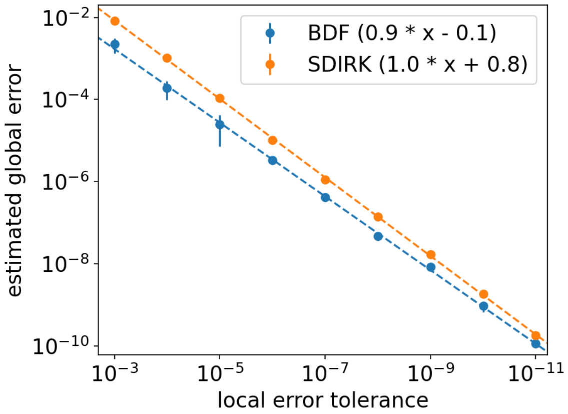

Figure S.2: **Approximate global numerical error of SDIRK and BDF solvers for a real-world ^{13}C-MFA model.** Mean approximated global numerical error $e_{num,ref}$ for the model Syn_a and common ($10^{-3} - 10^{-6}$) and high-accuracy tolerances ($10^{-6} - 10^{-11}$). Bars indicate standard deviations over random 10 parameter sets. The SDIRK reference solution is calculated with $tol_{rel} = 10^{-12}$ and $tol_{abs} = 10^{-15}$.

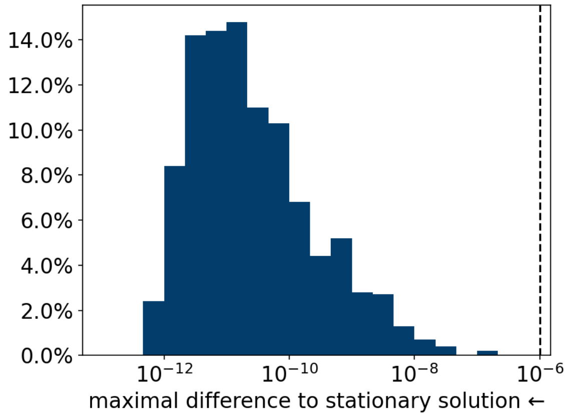

Figure S.3: `13CFLUX(v3)` **provides consistent IST and INST solutions.** Worst-case differences between numerical IST and INST solutions, measured according to Eq. (S13) for the EC_a model and 1,000 random parameter sets. INST IVPs are solved using the BDF solver with $tol_{rel} = 10^{-6}$ (indicated by the vertical dashed line) and $tol_{abs} = 10^{-9}$.

of the labeling systems in `13CFLUX(v3)` is consistent and remains numerically accurate and reliable for very large integration time windows.

S.2.4 Scalability of `13CFLUX(v3)`

In this section we investigate how the simulation runtime scales with increasingly complex measurement configurations and multiple datasets. All runtimes are computed with a single core of an AMD EPYC 9334 CPU.

S.2.4.1 Simulation of cumomer- and EMU-based labeling systems in `13CFLUX(v3)`

The simulation runtimes of the IST and INST labeling systems in Eq. (S2) and Eq. (S1) depend on the selected state space representations and their essential (i.e., dimension-reduced) dimensions (see Table S.1 in SI Section S.1.2). Exemplarily, here we benchmark the runtimes of the simulations of `13CFLUX(v3)` IST and INST of the essential cumomer and EMU representations for the *E. coli* model EC (see SI Section S.8.2 for details). For this, we use two configurations, a mass spectrometry (MS) from literature (EC_a) and a representative tandem mass spectrometry (MS/MS) configuration. For each of

these two measurement configurations, we create 34 systems of essential customer and EMU dimensions through measurement subsampling. For each of these systems, we benchmark the simulation runtime for 1,000 randomly generated parameter sets.

Figure S.4 shows a bar chart of the essential cumomer and EMU simulation times for the subsampled MS and MS/MS configurations. For MS measurement configurations, EMUs are significantly faster to simulate than cumomers for both, IST and INST labeling systems. This is due to the superior dimension-reduction capability of the essential EMU state-space formulation (Weitzel et al., 2007). However, cumomer-based labeling systems are faster to simulate for MS/MS configurations, despite achieving similar state-space dimension reduction factors as EMU-based systems. This is because the vector-shaped form of cumomer systems is cheaper to simulate than the matrix-shaped EMU systems (see also Figure S.5). These results underscore the importance of choosing the most efficient labeling state representation for a given measurement configuration to achieve fast simulations, as implemented in `13CFLUX(v3)`.

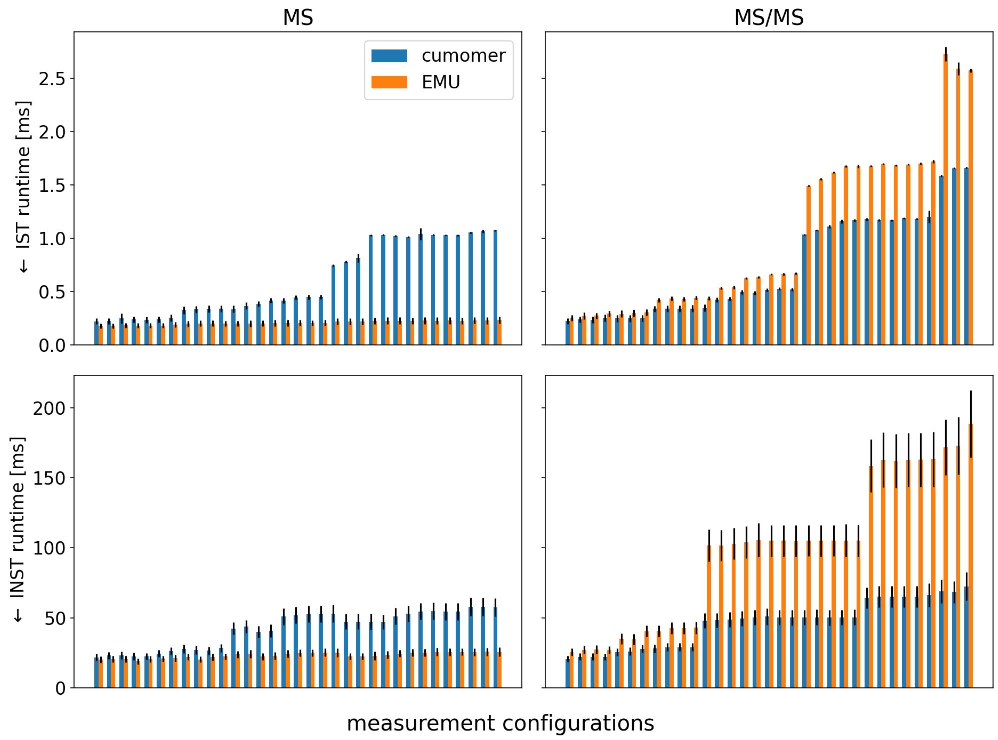

Figure S.4: **Whether cumomer- or EMU-based labeling systems are faster to simulate depends on the measurement configuration.** Mean IST and INST simulation times for the EC model and various MS and MS/MS measurement configurations for 1,000 parameter sets each. Bars indicate the standard deviation. IST (upper row) and INST (lower row) mean runtimes in milliseconds for essential cumomer (blue) and EMU (orange) labeling state representations.

In Figure S.5, we plot the simulation times for the subsampled MS and MS/MS configurations over their the essential cumomer and EMU dimensions. We observe an almost perfect correlation for both IST and INST labeling systems, with Pearson coefficients of > 0.99 in each case. As expected, solving the EMU labeling systems is more costly than solving the cumomer labeling systems for the same essential system dimensionality. This is because cumomer-based labeling states are scalars, whereas EMU-based labeling states are vectors, and the underlying labeling systems have more unknowns to solve for.

Eventually, the most beneficial state-space representation is determined by the specific analytical method (MS - EMU, MS/MS - cumomer), while the scaling behavior within a given analytical measurement setting depends on the particular combination of labeling system structure and measurement configuration.

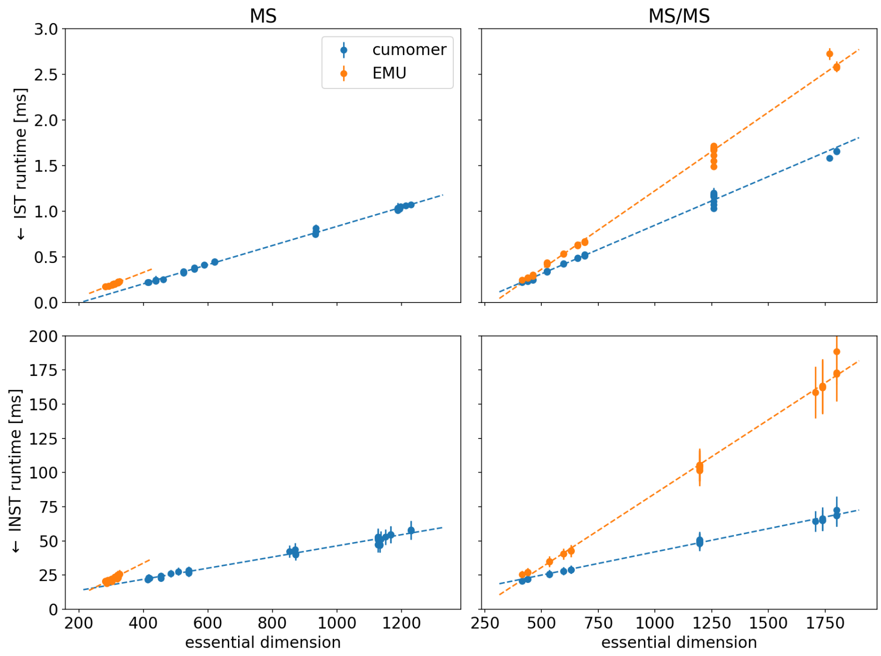

Figure S.5: **Cumomer-based simulations are generally faster than EMU-based simulations when considering the same essential system dimensions.** Mean IST and INST simulation times for the EC model with different MS and MS/MS configurations and random 1,000 parameter sets each. Bars indicate the standard deviation in simulation time. The relationship between essential cumomer/EMU labeling system dimensions and simulation times is highly correlated (Pearson coefficients > 0.99), pointing to `13CFLUX(v3)`'s perfect scaling behavior. The scaling behavior is specific to the type of measurement configuration.

S.2.4.2 Efficient simulation of datasets from multiple labeling experiments

The swift computation of parameter sensitivities as a solution of the IVPs in Eqns. (S3) and (S4) is crucial for all steps of the ^{13}C-MFA workflow where Jacobian matrices are required. One example is the Fisher information matrix (FIM) from which the parameter covariance matrix is derived. Both matrices are central to experimental design (ED) (Beyß et al., 2021). The classic approach to isotope tracer ED is to explore the information content of all possible combinations of selected isotope tracers, which are commonly calculated sequentially (Möllney et al., 1999). It is straightforward to see that, using a similar approach as described in SI Section S.1.3, we can reformulate the sequential ED calculation resembling Eq. (S6). The reformulation enables to parallelize ED evaluations in `13CFLUX(v3)`, either as a single one-shot evaluation or as a multiple-shot approach, split up into several evaluation batches with an adjustable size.

For the EC_a model (see SI Section S.8.2), we compare the runtimes of the new batch-wise evaluation approach with those of the traditional sequential evaluation in Eq. (S5)). The results in Figure S.6 show that employing the batch-wise formulation reduces runtimes by a factor of about 20 (batch size = 75). Consequently, using the batch-wise approach in `13CFLUX(v3)` to calculate EDs allows more tracers to be considered or a finer grid to be explored within a given computational budget, compared to the conventional sequential approach. This improvement unlocks large-scale ED calculations.

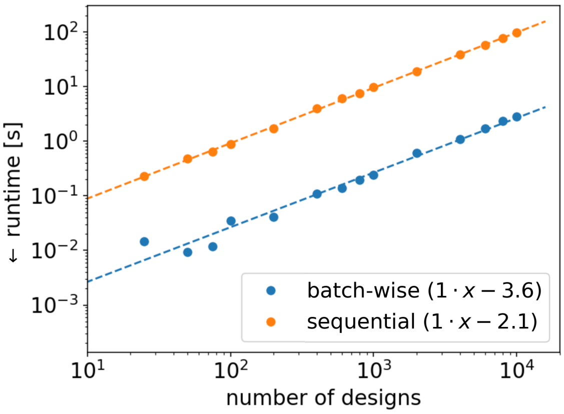

Figure S.6: **Batch-wise ED simulation of labeling systems is one order of magnitude faster than the traditional sequential approach.** Comparison of IST sensitivity system runtimes for sequential and batch-wise approaches. For the *E. coli* model EC_a the batch-wise approach scales consistently better than computing sensitivities sequentially.

S.3 13CFLUX(v3) implementation details

S.3.1 Software statistics

The 13CFLUX(v3) code consists of 132 files, most of which are written in C++ and Python. 13CFLUX(v3) is built using CMake (https://cmake.org/download/). The breakdown of the lines of code (LOC) taken for a snapshot of the repository is given in Table S.2. Numbers are generated with `cloc . --exclude-dir=docs,extern,.readthedocs.yaml --exclude-ext=fml,xml,md,xslt` to not include documentation and examples.

Language	LOCs	files
C++	14,062	108
Python	1,763	10
CMake	314	7
YAML/TOML	272	4
Dockerfile	90	3

Table S.2: Number of code lines of 13CFLUX(v3) resolved by programming language, quantified using cloc (Danial, 2021). The numbers refer to versioned code at commit 43e0ef34f9a432f061b9790c52b085be4aa8ec5b of the software repository.

S.3.2 Interoperation of 13CFLUX(v3) with third-party libraries

Interoperability is a key quality of the 13CFLUX(v3) software. It allows the software to work seamlessly with other complementary tools, extending its core features. 13CFLUX(v3) communicates with third-party libraries via its carefully designed application programming interface (API), which provides a standardized interface for exchanging information bidirectionally. For example, the simulator object generated for a specific model is accessible and usable by other software tools. Also, embedding functionalities of third party tools into 13CFLUX(v3) is possible. We here demonstrate the latter for the case of numerical optimizers, while an example for the former is given in SI Section S.6.1.

Numerical optimization computes best-fit parameters in ^{13}C-MFA by solving a linearly constrained nonlinear least-squares regression problem (Wiechert et al., 2015). These regression problems exhibit a wide range of difficulties attributed to the ill-conditionedness of the inverse problem (Theorell et al., 2017, Hadamard, 1902, Motulsky and Ransnas, 1987). Thus, there is no one-size-fits-all solution that handles all optimization tasks equally efficiently. Imbuilt in 13CFLUX(v3) is the open gradient-based numerical optimizer IPOPT (Interior Point Optimizer) (Wächter and Biegler, 2006), taken from the COIN-OR project (https://www.coin-or.org/) (Lougee-Heimer, 2003), to solve the linearly constrained nonlinear least-squares regression problem. However, when a good starting point is lacking or the parameter domain is to be explored, a gradient-free optimizer may be a better choice. In such situations, 13CFLUX(v3) makes it easy to use community optimizers and benefit from existing specialized algorithms and the continuous development of new, tailored ones. Here, we demonstrate how easy it is to replace the built-in IPOPT optimizer with a third-party one. In this example, we use the standard SciPy optimizer scipy.optimize.minimize (Virtanen et al., 2020).

```python
import scipy.optimize as optimize
ineq_sys = simulator.parameter_space.inequality_system
result = optimize.minimize(simulator.compute_loss, starting_point,
                           jac=simulator.compute_loss_gradient,
                           constraints=[optimize.LinearConstraint(ineq_sys.matrix,
                                                                  ub=ineq_sys.bound,
                                                                  keep_feasible=True)],
                           method="trust-constr")
```

S.3.3 Expressive error messages

Coding errors are unavoidable when creating ^{13}C-MFA evaluation workflows, particularly when new models or third-party software is involved (as described in SI Section S.3.2). Error messages and warnings are crucial to reducing the time spent on troubleshooting. However, detecting these errors and providing instructive solutions requires an extensive and careful analysis of possible reasons for those errors originating from technical (syntactic), contextual (logical), computational (numerical) and modeling (semantic) reasons. Therefore, we argue that providing expressive error messages is an important feature that distinguishes `13CFLUX(v3)` from other flux analysis tools.

`13CFLUX(v3)` provides more than 123 of such expressive messages (version 3.0.0a2). The following Table lists some examples.

**Error type**	**Case**	**Message**
semantic	Due to dimension reduction, all labeling states of a metabolite are eliminated from the cascaded labeling systems in Eq. (S1). In this case, the metabolite's pool size is to be eliminated from the IVP system, because it as no effect on the simulation outcome. This means that the pool size is under no circumstances identifiable from the given measurement setup and solving the respective part of the sensitivity system in Eq. (S3) is superfluous.	*Print warning* — Pool size of metabolite "A" is non-determinable given the measurement configuration, as it does not impact the simulation outcome. Consider fixing the pool size value in FluxML to reduce the ill-conditionedness of the inverse problem.
syntactic	Attempt to load a non-existing FluxML file.	*Throws ParseError* — Error with XML input source.
logic	Attempt to call labeling simulation or residual computation with infeasible model parameters, i.e., parameters that do not fulfill mass balances and/or violate inequality constraints.	*Throws ParameterError* — Free model parameters violate inequality constraints. Relax lower/upper bounds. If violations are very small, consider raising "parameter_space.constraint_violation_tolerance".
numerical	Attempt to solve the INST IVP in Eq. (S1) exceeds the number of maximal integration time steps, e.g. when very low error tolerances are requested.	*Throws MathError* — Operation "BDF (SUNDIALS) IVP solver" failed: Maximum number of steps (here 100,000) reached. Increase maximum number of solver time steps or relax solver tolerances.

S.4 Simulator collection

	Publication Date	Global (flux map) vs. Local (flux ratios)	State space			Simulation		Measurements			Workflows			Availability				
			Isotopomer	Cumomer	EMU	IST	INST	MS	Tandem-MS	NMR	Parameter fitting	Statistical analysis	Design of experiment	Plattfom independence	Commercial dependencies	Open code	License	Reference
tcaSIM, tcaCALC	1996	L	X			X		X		X	X	FS					?	https://doi.org/10.1002/mrm.1910360318 https://invivometabolism.org/tca.html
13C-FLUX	2001	G	X			X		X		X	X	FS	X				custom (academic/ commercial)	https://doi.org/10.1006/mben.2001.0188 https://13cflux.net/13cflux
13CFLUX2	2013	G		X	X	X		X	X	X	X	FS, PB PL	X				custom (academic/ commercial)	https://doi.org/10.1093/bioinformatics/bts646 https://13cflux.net/13cflux2
**13CFLUXv3**	**2025**	**G**		**X**	**X**	**X**	**X**	**X**	**X**	**X**	**X**	**FS, PB, PL, MCMC**	**X**	**X**		**X**	**AGPL**	https://13cflux.net/13cflux3
FiatFlux	2005	L				X		X			X	FS		X	MATLAB		custom (academic/ commercial)	https://doi.org/10.1186/1471-2105-6-209
SUMO FLUX	2016	L				X		X	X		X	PB		X	MATLAB	X	GPLv3	https://doi.org/10.1371/journal.pcbi.1005109 https://github.com/zamboni-lab/sumoflux
METRAN	2008	G			X	X		X			X	PL	X	X			?	https://doi.org/10.1074/jbc.M706494200 https://cheresearch.engin.umich.edu/mranton/m
OpenFlux	2009	G			X	X		X			X	PB, PL	X	X	MATLAB	X	GPLv2	https://doi.org/10.1186/1475-2859-8-25 https://sourceforge.net/projects/openflux
influx_s	2012	G		X	X	X		X		X	X	FS, PB		X		X	GPLv2	https://doi.org/10.1093/bioinformatics/btr716 https://github.com/sgsokol/influx
influx_si	2021	G		X	X		X	X		X	X	FS, PB		X		X	GPLv2	https://doi.org/10.1128/AEM.00719-21 https://github.com/sgsokol/influx
INCA	2014	G			X	X	X	X		1H	X	FS, PB, PL	X	X	MATLAB		custom (academic/ commercial)	https://doi.org/10.1093/bioinformatics/btu015 https://mfa.vueinnovations.com
INCA2	2022	G			X	X	X	X		X	X	FS, PB, PL	X	X	MATLAB		custom (academic/ commercial)	https://doi.org/10.1016/j.ymben.2021.12.009 https://mfa.vueinnovations.com
Open Mebius	2014	G			X	X	X	X			X	PL			MATLAB	X	?	https://doi.org/10.1155/2014/627014 https://en.metabolic-engineering.jp/page/softwar
mfapy	2021	G			X	X	X	X			X	PL	X	X		X	MIT	https://doi.org/10.1016/j.mec.2021.e00177 https://github.com/fumiomatsuda/mfapy
OpenFlux2	2014	G			X	X		X			X	FS, PB, PL	X	X	MATLAB	X	GPLv2	https://doi.org/10.1186/s12934-014-0152-x https://sourceforge.net/projects/openflux2
sysmetab	2016	G		X		X	X	X		X	X	FS, PB		X			?	https://doi.org/10.1109/TCBB.2016.2544299 https://gitlab.com/scilab/forge/sysmetab/
WUFlux	2016	G			X	X		X			X	PB		X	MATLAB	X	?	https://doi.org/10.1186/s12859-016-1314-0 https://tang.eece.wustl.edu/ToolDevelopment.htr
FluxPyt	2018	G			X	X		X			X	PB		X		X	BSD-3	https://doi.org/10.7717/peerj.4716 https://sourceforge.net/projects/fluxpyt
EMUlator	2019	G			X	X		X				none					?	https://doi.org/10.3389/fmicb.2019.00922
freeflux	2023	G			X	X	X	X			X	PB		X		X	GPLv3	https://doi.org/10.1021/acssynbio.3c00265 https://github.com/Chaowu88/freeflux
eiFlux	2022	G		X	X	X	X	X	X		X	PB		X	GAMS		?	https://doi.org/10.1371/journal.pcbi.1009831
CeCaFlux	2022	G			X	X	X	X			X	PL		X			?	https://doi.org/10.1093/bioinformatics/btac341 https://github.com/zhzhd82/CeCaFLUX
BayFlux	2024	G			X	X		X				MCMC		X		X	custom (free usage)	https://doi.org/10.1371/journal.pcbi.1011111 https://github.com/JBEI/bayflux

Table S.3: Common ^{13}C-MFA simulators, chronologically ordered by their first published version. Multiple versions are depicted as a group, indicated by black bars on the left side. FS: Fisherian statistics, PB: parametric bootstrap, PL: profile likelihoods, MCMC: Markov chain Monte Carlo, for details see (Theorell et al., 2017).

S.5 Simulator comparison

Next, we compare the simulation results of `13CFLUX(v3)` with those of three recent ^{13}C-MFA simulators capable of performing IST and INST simulations, namely `INCA` (version 2.3 with `MATLAB` R2022a), `FreeFlux` (version 0.3.6 with Python 3.10), and `influx_SI` (version 7.1 with Python 3.13 and R 4.4.1). Unless stated otherwise, we use default settings for the simulators. All three simulators rely on EMUs as labeling state space representation. In addition to numerical accuracy, we report simulation runtime benchmarks for the four simulators. Finally, the INST performance scaling behavior (accuracy versus runtime) of `13CFLUX(v3)` is compared with that of `INCA`. Because the simulators use slightly different natural ^{13}C abundances, these are set to 0.010816 for all simulators. All benchmarks are executed on a single core of an Intel Core i7-4790 CPU.

S.5.1 Comparison of simulated labeling states

For the simulator comparison, we use again the *E. coli* model (see SI Section S.8.2), with its original MS measurement configuration (`EC_a`). We set the model parameters to the flux values $\mathbf{v}$ reported in the `INCA` v2.3 example `demo/ecoli/ecoli.m`, which we hitherto denote *nominal fluxes*. We simulate the IST variant of the `EC_a` model and calculate the difference between the simulated labeling data of the three simulators – `freeflux`, `INCA`, and `influx_si` – and that of `13CFLUX(v3)`according to

$$e_{y,*,\texttt{13CFLUX(v3)}}(\mathbf{v}) = |y_*(\mathbf{v}) - y_{\texttt{13CFLUX(v3)}}(\mathbf{v})| \tag{S14}$$

where y denotes a single simulated measurement and the subscript $*$ represents one of the three simulators.

Absolute differences $e_{y,*,\texttt{13CFLUX(v3)}}$ from Eq. (S14) are shown in Figure S.7a. The numerical solutions of `13CFLUX(v3)` and `INCA` differ by no more than $4.2 \cdot 10^{-4}$ (on average, $1.2 \cdot 10^{-4}$). We attribute this difference to the conversion between the different flux coordinate systems used by the simulators (`INCA` uses the forward/backward flux coordinate system, `13CFLUX(v3)` the free net/exchange flux coordinate system). Nonetheless, the maximum difference in the solutions is one order of magnitude below the typical measurement standard deviation of $4 \cdot 10^{-3}$ and can therefore be considered negligible. However, compared to the results of `13CFLUX(v3)` and `INCA`, `freeflux` and `influx_si` show significantly larger deviations of up to $1.1 \cdot 10^{-2}$ (average $2.7 \cdot 10^{-3}$) and $5.8 \cdot 10^{-2}$ (average $2.0 \cdot 10^{-3}$), respectively.

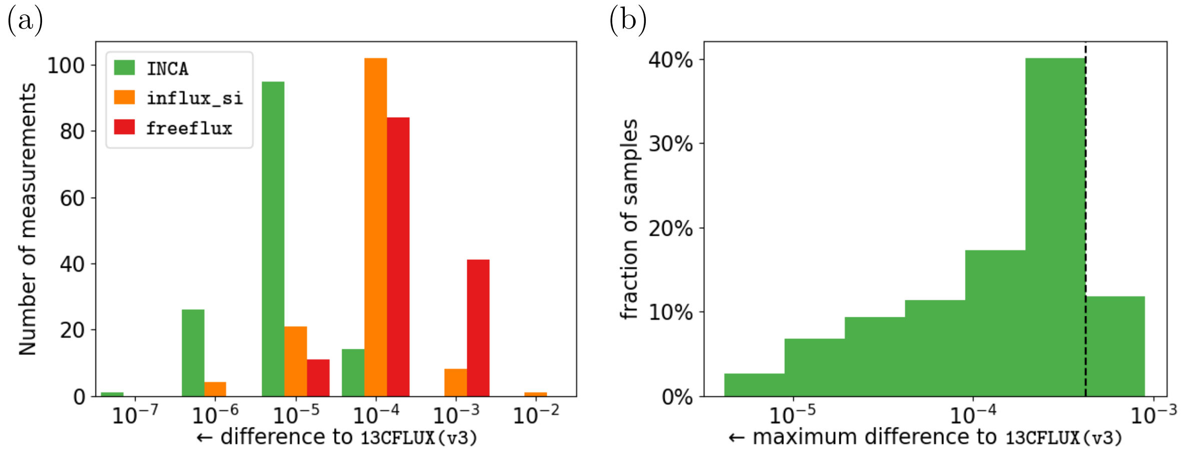

Figure S.7: **Simulation differences between SOTA ^{13}C-MFA tools for the `EC_a` model**. (a) **IST ^{13}C-MFA**. `13CFLUX(v3)` and `INCA` show good agreement (<4.2e-4 worst-case deviation); differences between `13CFLUX(v3)` and `influx_si` are one order of magnitude larger (<4.4e-3). `freeflux` shows differences to the `13CFLUX(v3)` simulation of up to 0.011, which is in the order of the measurement error. (b) **INST ^{13}C-MFA.** Results for `13CFLUX(v3)` and `INCA` are in good agreement. For almost 90% of the samples the maximum difference is smaller than that of the IST case (a). The maximum absolute difference is $7.4 \cdot 10^{-4}$. `13CFLUX(v3)` uses the BDF solver with $tol_{rel} = 10^{-6}$ and $tol_{rel} = 10^{-9}$.

For the INST case, the solution process of the associated labeling system is more sophisticated, and solution performances depend on flux and pool size values (see SI Section S.2.3.1). Systems of varying

difficulty are generated by augmenting the nominal fluxes with 1,000 random pool size configurations ($\mathbf{X}_j$ same as in SI Section S.2.3.3). Due to limitations of `freeflux` and `influx_si` in terms of solution performance (see below), we are required to restrict our comparison to `13CFLUX(v3)` and `INCA`.

For every parameter set, we compute the maximum absolute difference between the simulated labeling data vectors $\mathbf{y}(t)$ produced by the two simulators at the observed time points t_i in the set of measurement time points

$$e_{\mathtt{INCA,13CFLUX(v3)}}(\mathbf{v}, \mathbf{X}_j) = \max_{1 \le i \le N} \|\mathbf{y}_{\mathtt{13CFLUX(v3)}}(t_i, \mathbf{v}, \mathbf{X}_j) - \mathbf{y}_{\mathtt{INCA}}(t_i, \mathbf{v}, \mathbf{X}_j)\|_\infty, \; j = 1(1)1,000 \qquad \text{(S15)}$$

Both tools use the EMU state-space representation with solver tolerances set to $tol_{rel} = 10^{-6}$ and $tol_{abs} = 10^{-9}$.

The distribution of differences between the results of the two simulators, $e_{\mathtt{INCA,13CFLUX(v3)}}$ from Eq. (S15), is shown in Figure S.7b. For about 90% of the parameter sets, the difference in terms of $e_{\mathtt{INCA,13CFLUX(v3)}}$ is smaller than the largest IST difference, $e_{y,\mathtt{INCA,13CFLUX(v3)}}$, while the maximum absolute difference in the simulated labeling states in the parameter set is $7.4 \cdot 10^{-4}$. Consequently, the differences are in the same order of magnitude as in the IST case.

In conclusion, `INCA` and `13CFLUX(v3)` deliver comparable simulated labeling states for IST and INST with small differences that are likely to be attributed to their use of different flux coordinate systems. The differences between `13CFLUX(v3)` and `freeflux`, as well as `influx_si`, are greater in the IST case, whereas they cannot be properly assessed in the INST case.

S.5.2 Performance benchmark

To compare the time to perform forward simulation using `13CFLUX(v3)` to that of `freeflux`, `INCA` and `influx_si`, we set-up a benchmark with the EC (IST and INST) and Syn (INST) models (see SI Section S.1.2 for details). Simulations with two different label measurement configurations are performed because these configurations significantly impact the dimension of the system (see Table S.1). In addition, INST run times are taken for three IVP solver tolerances, namely $tol_{rel} = 10^{-3}$ (low accuracy), $tol_{rel} = 10^{-6}$ (standard accuracy), and $tol_{rel} = 10^{-9}$ (high accuracy). In all cases, the absolute tolerance tol_{abs} is set to $10^{-3} \cdot tol_{rel}$. The simulation task is repeated 100 times with the same parameter set. The mean and standard deviation are reported.

The results are summarized in Table S.4.

Clearly, INST simulations take longer than IST simulations. For INST, the solver tolerance significantly affects the runtime (see also Figure 1B in the main text). A runtime comparison of EC and Syn model variants reveals that, in addition to the network size (i.e., the number of metabolites and reactions) the effective dimensions of the dimension-reduced state spaces (see Table S.1) also play an important role.

The most important observations regarding the simulators are:

1. `freeflux` does not provide access to the IVP solver step-size, the solver tolerance is not adaptable, and the delivered simulation accuracy remains elusive.
2. `influx_si` has no automated step-size control, and the inbuilt setting fails to solve the system at higher accuracies.
3. `13CFLUX(v3)` outperforms `freeflux`, `INCA`, and `influx_si` in all settings.

S.5.3 Parameter variation

We quantify the effect of varying pool sizes on INST simulation times for the *E. coli* model EC_a. 1,000 random pool size parameter sets from Section S.2.3.3 are used for simulations with both the `13CFLUX(v3)` and `INCA` simulators. The tolerances for both simulators are set to $tol_{rel} = 10^{-6}$ and $tol_{abs} = 10^{-9}$. Each parameter set is calculated five times, and the mean value of the runtime is recorded. Mean runtime distributions are are shown in Figure S.8.

For `13CFLUX(v3)`, the runtime ranges from 15 ms to 207 ms (median 92.5 ms). For `INCA`, the median runtime is a factor of 6 larger (582 ms), and its distribution range is approximately 15 times larger (from 258 ms to 3,246 ms) than that of `13CFLUX(v3)`.

In conclusion, `13CFLUX(v3)` has on average shorter simulation times that are more robust with respect to parameter variations. While the variation in IST simulation times over different flux sets is nearly negligible, the runtime for INST strongly depends on the model parameters. Therefore, when benchmarking INST runtimes different parameter sets must be considered.

	Model	accuracy (tol_{rel})	13CFLUX(v3)†	freeflux*	INCA	influx_si**
INST	EC_a	10^{-3}	40.95 ± 0.81	214.17 ± 1.38	296 ± 123	225.07 ± 18.62
		10^{-6}	113.51 ± 3.60	N/A	2.790 ± 145	N/A
		10^{-9}	334.87 ± 12.85	N/A	$43{,}397 \pm 237$	N/A
	EC_b	10^{-3}	20.17 ± 0.80	188.43 ± 30.60	207 ± 66	57.55 ± 12.31
		10^{-6}	54.89 ± 0.50	N/A	759 ± 85	N/A
		10^{-9}	159.13 ± 3.78	N/A	$15{,}371 \pm 399$	N/A
	Syn_a	10^{-3}	32.54 ± 1.42	221.47 ± 1.85	199 ± 89	
		10^{-6}	96.58 ± 4.85	N/A	810 ± 157	
		10^{-9}	278.50 ± 7.37	N/A	$35{,}937 \pm 388$	
	Syn_b	10^{-3}	24.46 ± 0.38	161.94 ± 0.73	147 ± 47	
		10^{-6}	67.87 ± 2.77	N/A	548 ± 62	
		10^{-9}	201.35 ± 5.80	N/A	$16{,}359 \pm 315$	
IST	EC_a		0.45 ± 0.01	15.25 ± 0.08	$0^{\#}$	6.71 ± 2.48
	EC_b		0.26 ± 0.02	6.93 ± 0.14	$0^{\#}$	2.58 ± 0.40

Table S.4: **Simulator performance comparison.** Reported simulation times in [ms] are the mean ± standard deviation of 100 runs. N/A indicates that the simulator is not able to compute the desired configuration or cannot be configured as indicated. † EMU state-space representation. * `freeflux` has no possibility to influence the IVP solver tolerance. Therefore, runtimes are reported in the 10^{-3} accuracy row, although the achieved accuracy likely deviates. ** The IVP solver of `influx_si` has no automated step-size control. By manually adapting the step-size, a numerical accuracy of roughly 10^{-3} is achieved. When testing smaller step-sizes, the IVP solver generates numerically instable results. $^\#$ Due to limited runtime measurement resolution, IST runtimes are often reported to be 0.

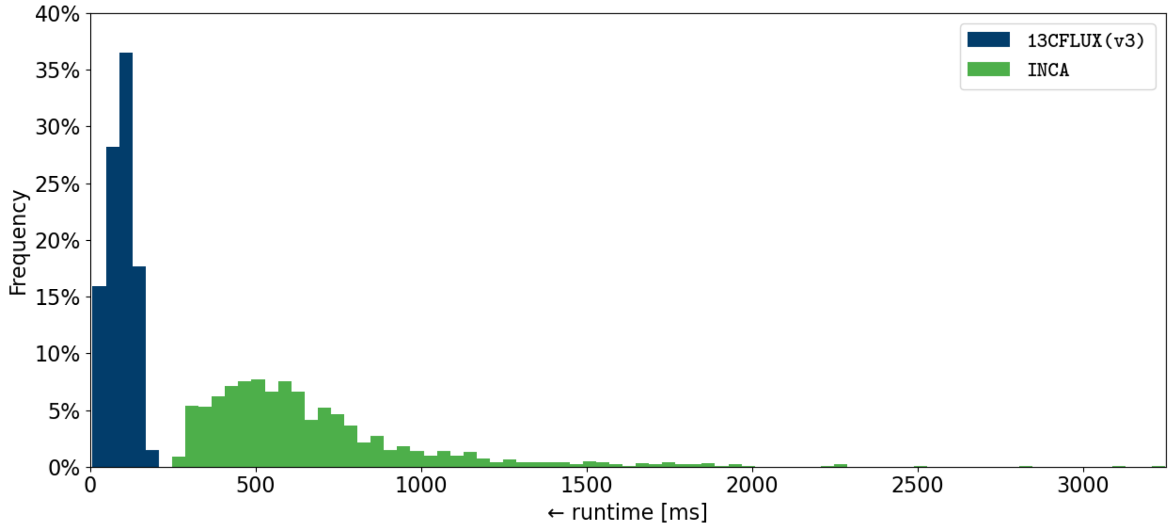

Figure S.8: **INST runtime variation for different sets of pool sizes.** `13CFLUX(v3)` shows a narrow distribution pointing to a very efficient step-size control.

S.5.4 Scalability of INST simulations

Finally, we compare the performance trade-off between simulation times and numerical accuracy for relative tolerances between $tol_{rel} = 10^{-2}$ and 10^{-12} (with $tol_{abs} = 10^{-3} \cdot tol_{rel}$). `13CFLUX(v3)` shows a linear correspondence between the logarithmic tolerances and the logarithmic runtimes in Figure S.9.

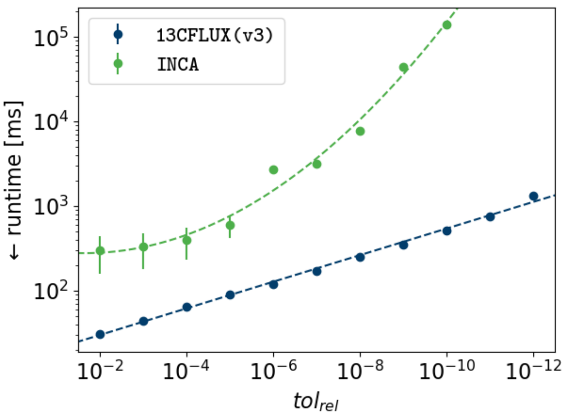

Figure S.9: `13CFLUX(v3)` **INST log runtimes scale linearly with decreasing log solver tolerances, whereas those of `INCA` scale quadratically.** Log-log plot of the mean runtimes (dots) taken for the EC_a model, with error bars originating from 100 repetitions. Linear and quadratic regression lines of $log_{10}(runtime)$ for `13CFLUX(v3)` (blue: $-0.1575 \cdot log_{10}(tol_{rel}) + 1.1582$) and `INCA` (green: $0.0392 \cdot (log_{10}(tol_{rel}))^2 + 0.1297 \cdot log_{10}(tol_{rel}) + 2.5504$), respectively.

The scaling behavior of `INCA` runtimes is comparable for relative tolerances of 10^{-2} and 10^{-5} (with an offset of one order of magnitude in runtimes). However, they grow quadratically on the logarithmic scale with decreasing (log) tolerances. For `13CFLUX(v3)`, an increase in relative tolerance of one order of magnitude requires approximately 40% more computing time.

S.6 Novel application: Bayesian INST ^{13}C-MFA

The Bayesian ^{13}C-MFA approach has several advantages over the parameter fitting based on optimization (Theorell et al., 2024), which still dominates the field. Most notably, the Bayesian approach provides a richer understanding of the probability of plausible parameter values and their dependencies through the *posterior probability distribution*. Analyzing the posterior distribution enables the detection of non-identifiable parameters and the examination of parameter correlations, offering a holistic understanding of parameter uncertainty. This is particularly appealing for INST ^{13}C-MFA, where the correlation between fluxes and pool sizes may be quite informative despite the fact that the single parameters may not be practically identifiable.

To estimate the posterior distribution of model parameters, their prior distribution, which captures existing knowledge, is combined with their likelihood in view of the data (Theorell et al., 2017). Stochastic Markov chain Monte Carlo (MCMC) methods approximate the posterior distribution of the fluxes by constructing a Markov chain that generates thousands to millions of random samples (simulations) (Brooks et al., 2011). These methods have strong theoretical underpinnings and asymptotic convergence guaranties to the true posterior under relatively mild conditions.

For IST ^{13}C-MFA, the Bayesian approach to parameter estimation has been described several times; see, for instance, Kadirkamanathan et al. (2006), Theorell et al. (2017), Backman et al. (2023), Hogg et al. (2023). However, the Bayesian approach for INST ^{13}C-MFA has not yet been attempted before. One reason is that, for MCMC to converge, many samples must be drawn (in the order of $10^6 - 10^9$), making fast simulation crucial. On the other hand, the parameter space to be sampled has complex polytopic geometry, which requires the construction of tailored Markov chains that efficiently traverse these spaces (Jadebeck et al., 2023).

To unlock Bayesian INST ^{13}C-MFA, we combine `13CFLUX(v3)` with the highly optimized polytope sampling tool `hopsy` (Paul et al., 2024). `hopsy` relies on the high-performance polytope sampling library `HOPS`, which is tailored toward efficiently explore the complex parameter spaces underlying ^{13}C-MFA models (Jadebeck et al., 2021).

S.6.1 Efficient MCMC sampling using 13CFLUX(v3)

`13CFLUX(v3)` provides the function `run_non_uniform_sampling` to facilitate MCMC sampling of the model parameters. To benefit from state-of-the-art MCMC algorithms, `13CFLUX(v3)` uses the MCMC sampling platform `hopsy`. High performance sampling analyses are maintained through the use of `numpy arrays`, the de facto standard for multidimensional data in Python, for communication. The expressive API of `13CFLUX(v3)` enables integration with `hopsy` in just a few lines of Python code:

```
1   def run_non_uniform_sampling(simulator, num_samples: int, starting_point: np.ndarray,
2                                bounds: Dict[str, Tuple[float, float]],
3                                num_chains: int,
4                                proposal: hopsy.PyProposal,
5                                random_seed: int,
6                                **kwargs):
7       problem = hopsy.Problem(ineq_constr_matrix, ineq_constr_bound, HopsyModel(simulator))
8       if starting_point is None:
9           problem.starting_point = hopsy.compute_chebyshev_center(problem)
10      else:
11          problem.starting_point = starting_point
12
13      mcs, rngs = hopsy.setup(problem, random_seed, n_chains=num_chains, proposal=proposal)
14      _, samples = hopsy.sample(mcs, rngs, n_samples=num_samples, n_procs=num_chains, **kwargs)
15
16      return samples
```

In line 7, the `13CFLUX(v3)` simulator object is encapsulated by a `x3cflux.HopsyModel` object, which is part of the `13CFLUX(v3)` API. The `x3cflux.HopsyModel` object manages the translation from `13CFLUX(v3)` (`compute_loss`) to `hopsy` (`log_density`) according to

```
class HopsyModel:
    def __init__(self, simulator):
        self.simulator = simulator

    def log_density(self, x):
        return -0.5 * self.simulator.compute_loss(x)
```

Line 5-6 encodes the translation between the `13CFLUX(v3)` (C++) and `hopsy` (Python). Specifically, the code lines define the likelihood log-density in `hopsy` as the negated and halved residuals computed by `13CFLUX(v3)`. This code exemplifies, how the functionality of `13CFLUX(v3)` is extended to perform efficient MCMC-based statistical analyses.

S.6.2 Application

We now perform Bayesian INST ^{13}C-MFA using `13CFLUX(v3)`, where we select the *Synechocystis* model Syn_a as use case (see SI Section S.8 for details). To be able to assess the quality of the posterior probabilities provided by Bayesian inference, we rely on a synthetic dataset, simulated for realistic parameters, which are perturbed according to typical measurement errors (`syn_perturbed.fml`). For the pool sizes normal prior distributions are formulated, exchange fluxes are constraint by an upper bound of 100. Four Markov chains are then run from independent dispersed initial starting points. We employ parallel tempering (Geyer, 1991) to tackle potential multi-modalities of the posterior distribution. The chains are run for 2,400,000 samples, which were reduced to 24,000 samples by using thinning (thinning factor of 100).

The computational workflow is set up in the Python script (`S6_1-INST-mcmc.py`). Since the Bayesian analysis of INST ^{13}C-MFA is compute intense, we provide a Docker container containing the workflow script, `13CFLUX(v3)` and `hopsy`, which can be executed on a workstation or compute server. We recommend using Slurm (Slurm scripts are reproduced by `S6_0-create-SLURM-scripts.py`) to parallelize the sampling workflow across nodes. Alternatively, the samples can be reproduced on a single compute node using the command

```
docker run -v .:/task jugit-registry.fz-juelich.de/ibg-1/modsim/fluxomics/13cflux:latest \
        /task/run-sampling.sh
```

To check for the convergence of the (thinned) Markov chains, it is common to calculate the rank-normalized potential scale reduction factor (PSRF) or $\hat{R}$ value (Vehtari et al., 2021) and check whether its values for each parameter is below a threshold close to one.

As a final visual quality check, posterior predictive plots are generated by forward simulating a representative subset of the posterior samples.

Figure S.10 shows marginal and joint posterior distributions for selected parameters (net fluxes and pool sizes). The red crosses mark the ground truth solution. The posterior probability modes match the ground truth parameters well. The results reveal a non-normality of the marginal posterior distribution of the *sba.n* net flux, the fact that the *tal2.x* exchange flux is nearly non-identifiable, and that the net fluxes *co2in.n* and *gapdh.n* are correlated. Furthermore, the marginal posterior for the CO2 metabolite resembles its prior.

Figure S.11 shows exemplary posterior predictive plots. The posterior predictive labeling curves are in excellent agreement with the data and exhibit a low prediction variance. This indicates that the model perfectly matches the data, as expected for the synthetic case studied here.

In summary, by its interoperability with the MCMC toolbox `hopsy` and the portability enabled by Docker, `13CFLUX(v3)` unlocks convenient and computationally scalable Bayesian INST ^{13}C-MFA workflows.

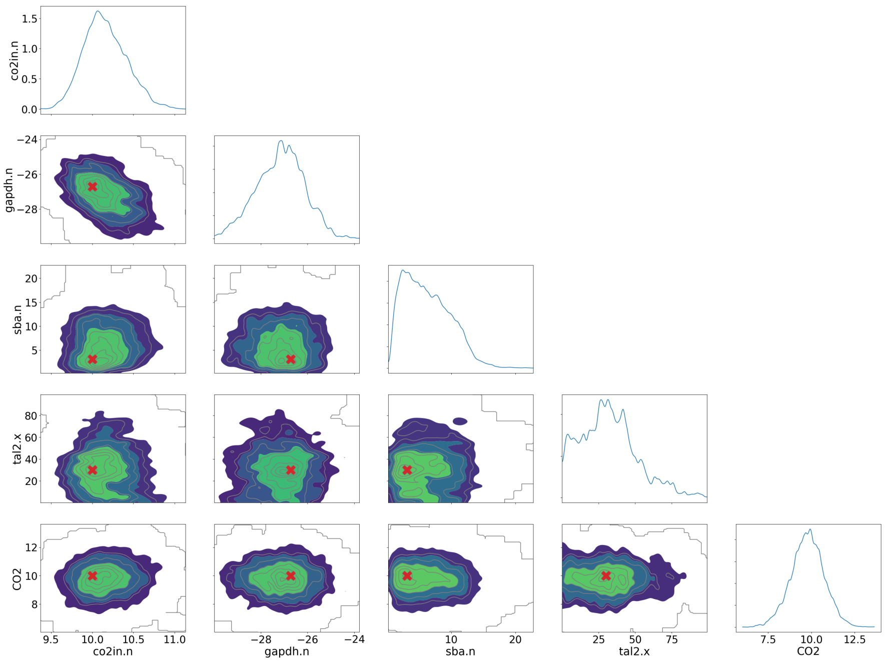

Figure S.10: **Marginal and 2D INST ^{13}C-MFA parameter posterior distributions for Syn_a model.** MCMC sample distributions are smoothed using kernel density estimation. The red crosses mark the ground truth values. For each free parameter the rank-normalized potential scale reduction factor $\hat{R}$ is below 1.05.

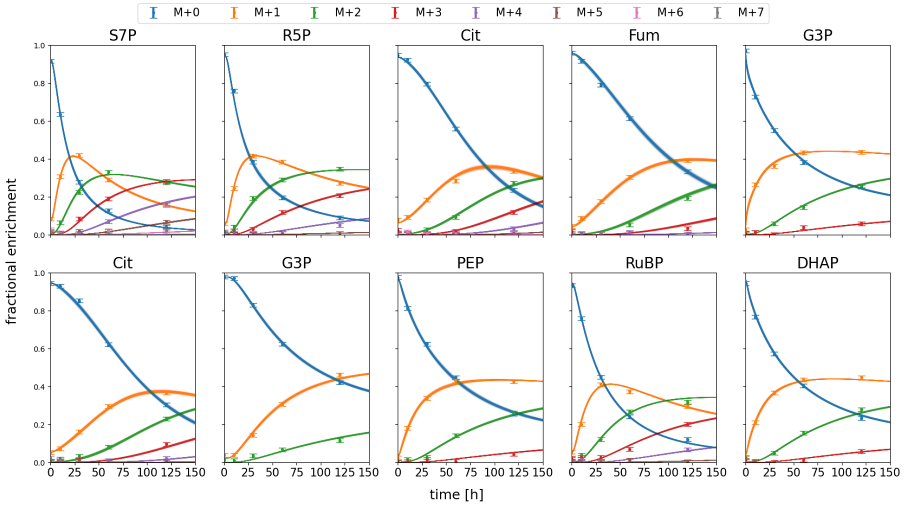

Figure S.11: **Posterior predictive plots for the Syn_a model.** 100 samples are used to simulate data (lines) and plotted with the data points (capped bars).

S.7 `Airflow`’ing `13CFLUX(v3)` production workflows

Scientific workflows delineate the sequence of computational steps involved in a flux analysis, such as executing a simulation of a ^{13}C-MFA network model or performing statistical inference on experimental datasets (Dalman et al., 2013). When formalized as a directed graph – in which nodes represent inputs and analysis tasks, and edges define the dependencies between them – such workflows offer a precise structural representation of the analysis pipeline performed to handle the task at hand. This formalization promotes both the reproducibility and repeatability of evaluations, which improves the overall scientific quality. Moreover, the representation facilitates workflow automation for routine analyses, where the same steps are applied over and over again. This automation reduces manual efforts and minimizes potential errors.

Apache `Airflow` is a powerful workflow orchestration platform that is actively maintained by a large user community (https://airflow.apache.org/). Lately, `Airflow` has also gained traction for MLops (machine learning operations), which involves deploying and maintaining machine learning models in production environments, proving an excellent choice for automating and monitoring software.

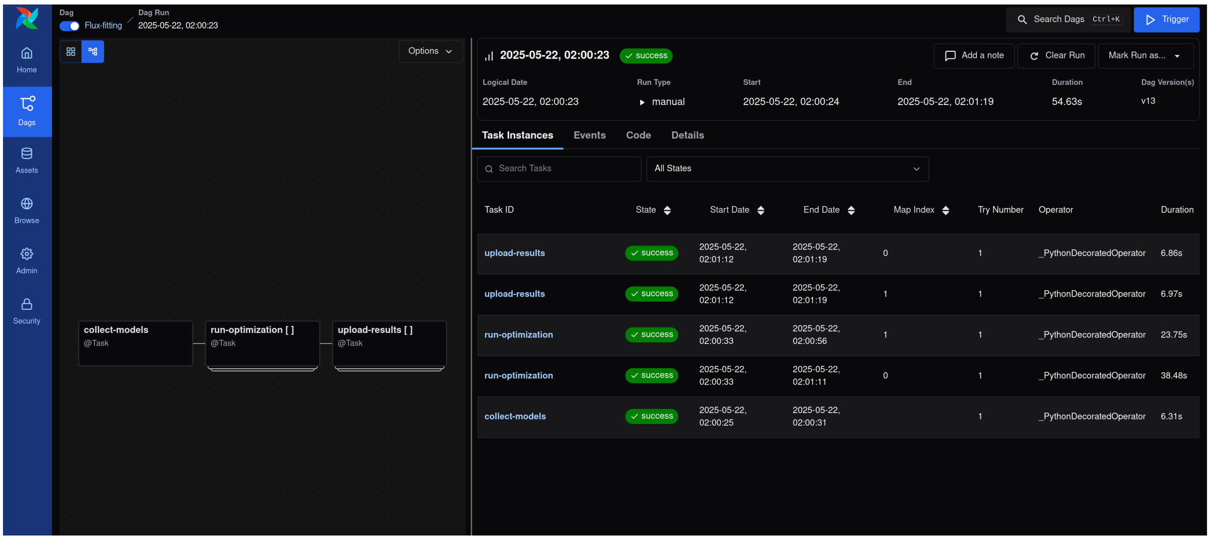

Figure S.12: `Airflow`DAG for flux fitting. `Airflow-3` provides a modern web user interface for managing and monitoring workflows. Two FluxML models are collected from the cloud storage, the dynamic task mapping creates two *run-optimization* and two *upload-results* tasks.

In `Airflow`, workflows are defined using DAGs (directed acyclic graphs). DAGs are set-up in Python and automatically visualized in the `Airflow` user interface (see Figure S.12). The nodes in a DAG represent tasks that need to be executed, and `Airflow` automatically computes their dependencies based on the provided workflow constraints, such as task A must be completed before task B. Tasks are executed by *executors*, which offer flexibility in terms of where and how to run a task. For example, computationally intensive tasks can be run on workstations, either natively or within Docker images, providing a well-defined compute environment. Additionally, `Airflow` provides monitoring and alerting capabilities, enabling process and status tracking of the workflow execution.

Figure S.12 shows an `Airflow` DAG for a simple ^{13}C-MFA fitting workflow, which is intentionally kept simple (excluding statistical tests, visualizations etc.). In the first workflow step, users upload their models to an S3 cloud storage device, the so-called S3 bucket. DAGs are defined to run on a schedule or triggered manually, as in this case. Once the flux fitting DAG is triggered, it collects the models from the S3 bucket. Using dynamic task mapping, the tasks to be run are adjusted to match the number of flux models. For each model, a multi-start optimization is run and the results as well as their associated model are uploaded to a separate S3 location, where they can be collected by users. Further useful tasks are e-mail notifications that inform users as soon as the workflow is completed.

The Python listing for this ^{13}C-MFA workflow is shown below. The code can be easily extended by adding steps such as statistical or visual quality controls. Note that in addition to an `Airflow` instance, an S3 server is required to execute this code.

```python
import boto3
import datetime
import logging
import json
import os
import tempfile
import x3cflux
from pathlib import Path
from airflow.sdk import DAG, task
from airflow.models import Variable
logger = logging.getLogger(__name__)

s3_id = Variable.get("s3_id")
s3_secret =  Variable.get("s3_secret")
s3_endpoint = Variable.get("s3_endpoint")
s3_bucket = "13CFLUX3"
remote_prefix = "models/"
results_prefix = "results/"

with (DAG(
        dag_id="Flux-fitting",
        start_date=datetime.datetime(2025, 1, 1),
        schedule="@daily",
)):
    @task(task_id="collect-models")
    def collect_models(id, secret, bucket, endpoint, remote_prefix=remote_prefix):
        session = boto3.session.Session()
        s3_client = session.client(
            service_name="s3",
            aws_access_key_id=id,
            aws_secret_access_key=secret,
            endpoint_url=endpoint,
        )
        response = s3_client.list_objects_v2(Bucket=bucket, Prefix=remote_prefix, Delimiter="/")
        fml_files = []
        for fml_file in response.get("Contents", []):
            if fml_file['Key'].endswith('.fml'):
                fml_files.append(fml_file['Key'])
        return fml_files


    @task(task_id="run-optimization")
    def run_optimization(fml_file, id, secret, bucket, endpoint, remote_prefix=remote_prefix):
        session = boto3.session.Session()
        s3_client = session.client(
            service_name="s3",
            aws_access_key_id=id,
            aws_secret_access_key=secret,
            endpoint_url=endpoint,
        )
        with tempfile.TemporaryDirectory() as tmp:
            os.mkdir(os.path.join(tmp, remote_prefix))
            local_name = os.path.join(tmp, fml_file)
            with open(local_name, "wb") as f:
                s3_client.download_fileobj(bucket, fml_file, f)
            simulator = x3cflux.create_simulator_from_fml(local_name)
            # Starts multi-fit from uniformly distributed points
            starting_points = x3cflux.run_uniform_sampling(simulator, 10_000)[0].T
            logger.info(f'running multistart optimization for {local_name}')
            optima, losses = x3cflux.run_multi_optimization(simulator, starting_points, num_procs=8)
            return {"fml_file": fml_file, "optima": optima.tolist(), "losses": losses.tolist()}


    @task(task_id="upload-results")
    def upload_results(optimization_result, id, secret, bucket, endpoint, remote_prefix=remote_prefix):
        session = boto3.session.Session()
        s3_client = session.client(
            service_name="s3",
            aws_access_key_id=id,
            aws_secret_access_key=secret,
            endpoint_url=endpoint,
        )
        fml_file = optimization_result['fml_file']
        model_name = Path(fml_file).stem
        json_name = f"{model_name}_optimization_result.json"
```

```
    with tempfile.TemporaryDirectory() as tmp:
        os.mkdir(os.path.join(tmp, remote_prefix))
        local_name = os.path.join(tmp, remote_prefix, json_name)
        with open(local_name, "w") as f:
            json.dump(optimization_result, f)
        logger.info(f'uploading {local_name}')
        # Moves fml file from models
        s3_client.upload_file(local_name, bucket, os.path.join(results_prefix, json_name))

        local_fml_name = os.path.join(tmp, fml_file)
        with open(local_fml_name, "wb") as f:
            s3_client.download_fileobj(bucket, fml_file, f)
        s3_client.upload_file(
            local_fml_name,
            bucket,
            os.path.join(results_prefix, os.path.basename(fml_file))
        )

        delete_response = s3_client.delete_object(
            Bucket=bucket,
            Key=fml_file
        )

# collects models to fit from s3
fml_files = collect_models(id=s3_id, secret=s3_secret, bucket=s3_bucket, endpoint=s3_endpoint)
#  runs optimization
optimization_results = run_optimization.partial(
    id=s3_id, secret=s3_secret, bucket=s3_bucket, endpoint=s3_endpoint
).expand(fml_file=fml_files)
#  uploads results
upload_results.partial(
    id=s3_id, secret=s3_secret, bucket=s3_bucket, endpoint=s3_endpoint
).expand(optimization_result=optimization_results)
```

S.8 Metabolic models

In this work, ^{13}C-MFA network models are utilized in different variations. In this section, most important facts about the base models are shortly summarized.

S.8.1 Linear pathway model

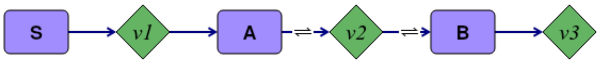

Figure S.13: Model of a linear pathway with one exchange flux.

The linear pathway model represents a linear reaction chain with the external metabolite S (substrate) and two internal metabolites (A, B). A and B are connected via a bidirectional reaction v_2 (see Figure S.13). All metabolites carry one (carbon) atom. With the pool size $B = 1$, the net fluxes $v_{1,net} = v_{2,net} = v_{3,net} = 1$, the exchange flux $v_{2,xch} = \tau$, and fully labeled input substrate, the labeling states of the metabolites A and B of this linear reaction chain coincide with the test ODE in SI Section S.2.3.1. Profile of the linear pathway model:

Reaction network: 3 metabolites (2 intracellular), 3 reactions (2 intracellular, 1 bidirectional)

Independent parameters: 1 net flux, 1 exchange flux, 2 pool sizes. We fix one pool size ((B)) and one net flux; thus only one unknown exchange flux and one unknown pool size remain.

Measurement configuration: 1 mass spectrometry (MS) measurement of B at 1 time point

Labeling system: 4 isotopomer states, 2 cumomer or EMU states, $K = 1$ cascade levels

S.8.2 *Escherichia coli* (EC)

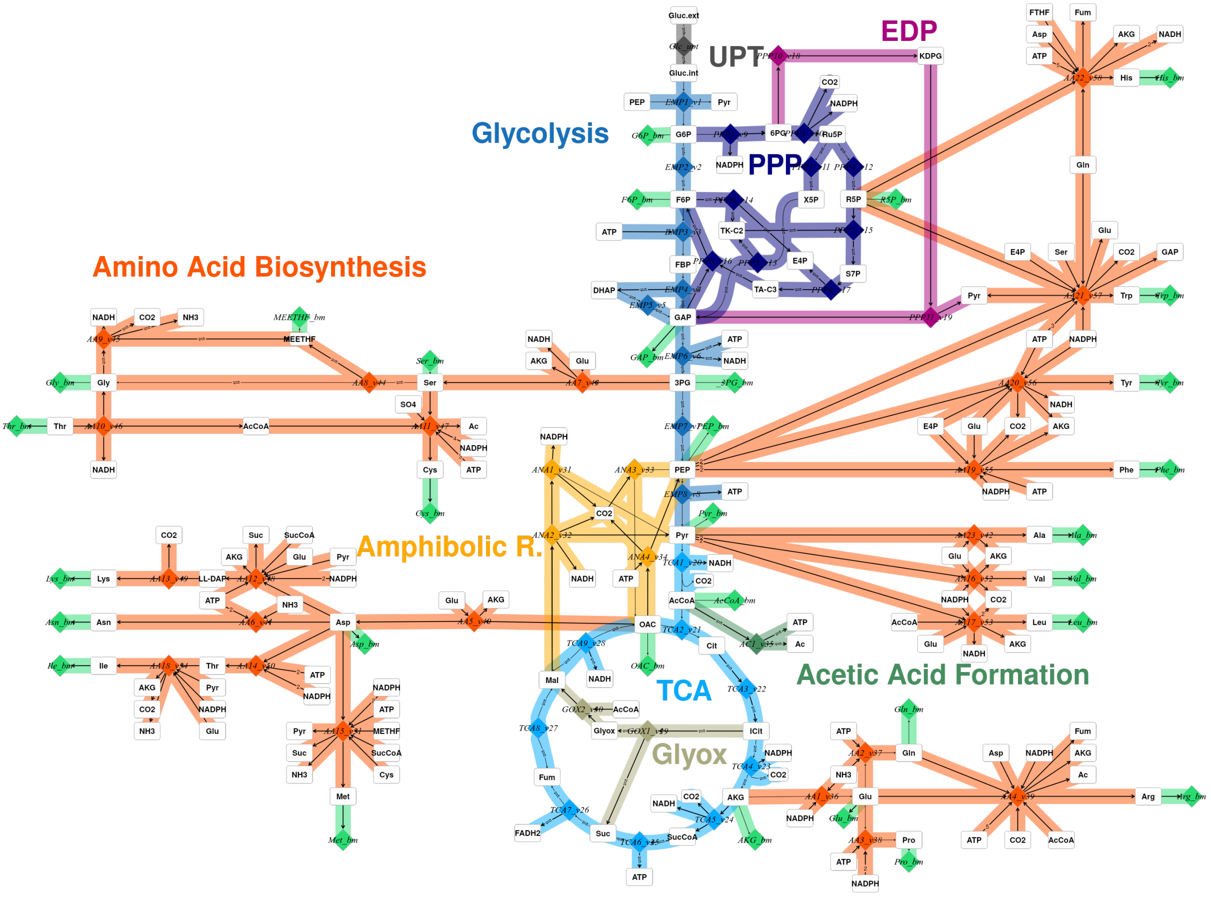

Figure S.14: **Network model of *E. coli*.** Throughout the text the model is referred to as EC.

EC is a model of the central carbon metabolism of *E. coli* including a detailed formulation of amino acid formation. The model is provided with the `INCA` software version 2.3 (`demo/ecoli/ecoli.m` gives rise to a variant for INST ^{13}C-MFA from which we have created an IST model variant). It comprises 100 reactions connecting 60 metabolites, features 4,858 isotopomers, and has 12/30/56 free model parameters (net fluxes/exchange fluxes/pool sizes). Two measurement configurations are available for both IST and INST ^{13}C-MFA: The original measurement configuration, termed EC_a, has 33 (partially doubled) MS measurements of amino acid fragments and intermediates, for IST taken at 1 and for INST taken at 9 equidistant measurement time points, resulting in a total of 120 and 1,161 independent labeling measurements for IST and INST, respectively. A second measurement configuration, termed EC_b, has one MS measurement of an Alanine (Ala) fragment at the same measurement time points as EC_a, resulting in a total of 2 and 18 independent labeling measurements for IST and INST, respectively.

S.8.3 *Synechocystis* (Syn)

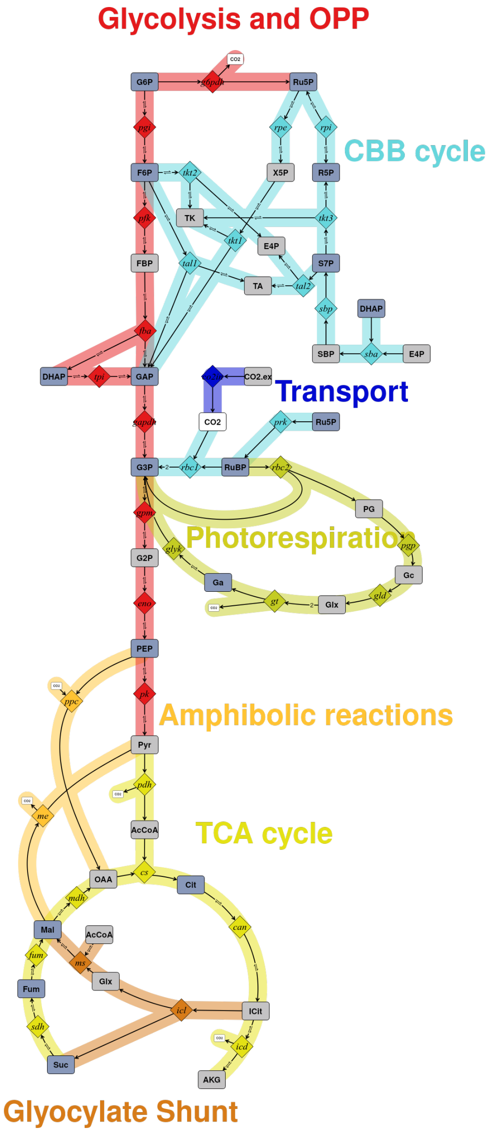

Figure S.15: **Network model of *Synechocystis*.** Throughout the text it is referred to as Syn.

Syn is a model of the central carbon metabolism of *Synechocystis*. The model is provided with the `freeflux` software (`models/synechocystis`), being a variant of INST ^{13}C-MFA. It comprises 57 reactions connecting 38 metabolites, features 992 isotopomers, and has 7/22/32 free model parameters (net fluxes/exchange fluxes/pool sizes). Two measurement configurations are available: the original measurement configuration, termed Syn_a, has 18 MS measurements of intermediates, taken at eight increasingly spaced measurement time points, resulting in a total of 590 independent labeling measurements. A second measurement configuration, termed Syn_b, has one MS measurement of glyceraldehyde-3-phosphate (G3P) at the same measurement time points as Syn_a, resulting in a total of 24 independent labeling measurements.

References

M. R. Antoniewicz, J. K. Kelleher, and G. Stephanopoulos. Elementary metabolite units (EMU): A novel framework for modeling isotopic distributions. *Metabolic Engineering*, 9(1):68–86, 2007. doi: 10.1016/j.ymben.2006.09.001.

T. W. H. Backman, C. Schenk, T. Radivojevic, D. Ando, J. Singh, J. J. Czajka, Z. Costello, J. D. Keasling, Y. Tang, E. Akhmatskaya, and H. Garcia Martin. BayFlux: A Bayesian method to quantify metabolic fluxes and their uncertainty at the genome scale. *PLOS Computational Biology*, 19(11): e1011111, 2023. doi: 10.1371/journal.pcbi.1011111.

M. Beyß, V. D. Parra-Peña, H. Ramirez-Malule, and K. Nöh. Robustifying experimental tracer design for ^{13}C-metabolic flux analysis. *Frontiers in Bioengineering and Biotechnology*, 9, 2021. doi: 10.3389/fbioe.2021.685323.

S. Brooks, A. Gelman, G. Jones, and X.-L. Meng. *Handbook of Markov Chain Monte Carlo*. London: Chapman and Hall/CRC, 2011. doi: 10.1201/b10905.

G. G. Dahlquist. A special stability problem for linear multistep methods. *BIT*, 3(1):27–43, mar 1963. doi: 10.1007/BF01963532.

T. Dalman, T. Dörnemann, E. Juhnke, M. Weitzel, W. Wiechert, K. Nöh, and B. Freisleben. Cloud MapReduce for Monte Carlo bootstrap applied to metabolic flux analysis. *Future Generation Computer Systems*, 29(2):582–590, 2013. doi: 10.1016/j.future.2011.10.007.

A. Danial. cloc: v1.92, 12 2021. URL `https://doi.org/10.5281/zenodo.5760077`.

C. J. Geyer. Markov Chain Monte Carlo maximum likelihood. In *Computing Science and Statistics: Proceedings of the 23 Symposium on the Interface*, pages 156–163. Interface Foundation of North America, 1991.

J. Hadamard. Sur les problèmes aux dérivées partielles et leur signification physique. *Princeton University Bulletin*, 13(4):49–52, 04 1902.

E. Hairer and G. Wanner. *Solving Ordinary Differential Equations II*, volume 14 of *Springer Series in Computational Mathematics*. Springer, Berlin, Heidelberg, 1996. doi: 10.1007/978-3-642-05221-7.

A. C. Hindmarsh, P. N. Brown, K. E. Grant, S. L. Lee, R. Serban, D. E. Shumaker, and C. S. Woodward. SUNDIALS: Suite of nonlinear and differential/algebraic equation solvers. *ACM Transactions on Mathematical Software (TOMS)*, 31(3):363–396, 2005. doi: 10.1145/1089014.1089020.

M. Hogg, E.-M. Wolfschmitt, U. Wachter, F. Zink, P. Radermacher, and J. A. Vogt. Bayesian ^{13}C-metabolic flux analysis of parallel tracer experiments in granulocytes: A directional shift within the non-oxidative pentose phosphate pathway supports phagocytosis. *Metabolites*, 14(1):24, 2023. doi: 10.3390/metabo14010024.

J. F. Jadebeck, A. Theorell, S. Leweke, and K. Nöh. HOPS: High-performance library for (non-)uniform sampling of convex-constrained models. *Bioinformatics*, 37(12):1776–1777, 2021. doi: 10.1093/bioinformatics/btaa872.

J. F. Jadebeck, W. Wiechert, and K. Nöh. Practical sampling of constraint-based models: optimized thinning boosts CHRR performance. *PLOS Computational Biology*, 19(8):e1011378, 2023. doi: 10.1371/journal.pcbi.1011378.

V. Kadirkamanathan, J. Yang, S. A. Billings, and P. C. Wright. Markov chain Monte Carlo algorithm based metabolic flux distribution analysis on *Corynebacterium glutamicum*. *Bioinformatics*, 22(21): 2681–2687, 2006. doi: 10.1093/bioinformatics/btl445.

C. P. Long and M. R. Antoniewicz. High-resolution ^{13}C metabolic flux analysis. *Nature Protocols*, 14 (10):2856–2877, 2019. doi: 10.1038/s41596-019-0204-0.

R. Lougee-Heimer. The common optimization interface for operations research: Promoting open-source software in the operations research community. *IBM Journal of Research and Development*, 47(1): 57–66, 2003. doi: 10.1147/rd.471.0057.

M. Möllney, W. Wiechert, D. Kownatzki, and A. A. de Graaf. Bidirectional reaction steps in metabolic networks. IV. Optimal design of isotopomer labeling experiments. *Biotechnology and Bioengineering*, 66(2):86–103, 1999. doi: 10.1002/(SICI)1097-0290(1999)66:2⟨86::AID-BIT2⟩3.0.CO;2-A.

H. J. Motulsky and L. A. Ransnas. Fitting curves to data using nonlinear regression: a practical and nonmathematical review. *The FASEB Journal*, 1(5):365–374, 11 1987. doi: 10.1096/fasebj.1.5.3315805.

K. Nöh, A. Wahl, and W. Wiechert. Computational tools for isotopically instationary ^{13}C labeling experiments under metabolic steady state conditions. *Metabolic Engineering*, 8(6):554–577, 2006. doi: 10.1016/j.ymben.2006.05.006.

A. Nordsieck. On numerical integration of ordinary differential equations. *Mathematics of Computation*, 16(77):22–49, 1 1962. doi: 10.1090/S0025-5718-1962-0136519-5.

R. D. Paul, J. F. Jadebeck, A. Stratmann, W. Wiechert, and K. Nöh. hopsy – a methods marketplace for convex polytope sampling in Python. *Bioinformatics*, 40(7):1–5, 2024. doi: 10.1093/bioinformatics/btae430.

A. Theorell, S. Leweke, W. Wiechert, and K. Nöh. To be certain about the uncertainty: Bayesian statistics for ^{13}C metabolic flux analysis. *Biotechnology and Bioengineering*, 114(11):2668–2684, 2017. doi: 10.1002/bit.26379.

A. Theorell, J. F. Jadebeck, W. Wiechert, J. McFadden, and K. Nöh. Rethinking ^{13}C-metabolic flux analysis – The Bayesian way of flux inference. *Metabolic Engineering*, 83:137–149, 2024. doi: 10.1016/j.ymben.2024.03.005.

A. Vehtari, A. Gelman, D. Simpson, B. Carpenter, and P.-C. Bürkner. Rank-normalization, folding, and localization: an improved $\widehat{R}$ for assessing convergence of MCMC. *Bayesian Analysis*, 16(2), 2021. doi: 10.1214/20-BA1221.

P. Virtanen, R. Gommers, T. E. Oliphant, M. Haberland, and T. Reddy et. al. SciPy 1.0: fundamental algorithms for scientific computing in Python. *Nature Methods*, 17(3):261–272, 2020. doi: 10.1038/s41592-019-0686-2.

A. Wächter and L. T. Biegler. On the implementation of an interior-point filter line-search algorithm for large-scale nonlinear programming. *Mathematical Programming*, 106(1):25–57, 2006. doi: 10.1007/s10107-004-0559-y.

M. Weitzel, W. Wiechert, and K. Nöh. The topology of metabolic isotope labeling networks. *BMC Bioinformatics*, 8:315, 2007. doi: 10.1186/1471-2105-8-315.

W. Wiechert and M. Wurzel. Metabolic isotopomer labeling systems. Part I: Global dynamic behavior. *Mathematical Biosciences*, 169(2):173–205, 2001. doi: 10.1016/S0025-5564(00)00059-6.

W. Wiechert, K. Nöh, and M. Weitzel. Metabolic isotopomer labeling systems. Part III: Path tracing. *Mathematical Biosciences*, 244(1):1–12, 2013. doi: 10.1016/j.mbs.2013.02.012.

W. Wiechert, S. Niedenführ, and K. Nöh. A primer to ^{13}C metabolic flux analysis. In J. Villadsen, editor, *Fundamental Bioengineering*, pages 97–142. Wiley-VCH Verlag GmbH & Co. KGaA, Weinheim, Germany, 2015. doi: 10.1002/9783527697441.ch05.

J. D. Young, J. L. Walther, M. R. Antoniewicz, H. Yoo, and G. Stephanopoulos. An elementary metabolite unit (EMU) based method of isotopically nonstationary flux analysis. *Biotechnology and Bioengineering*, 99(3):686–699, 2008. doi: 10.1002/bit.21632.
